# MuSK is a substrate for CaMK2β but this interaction is dispensable for MuSK activation *in vivo*

**DOI:** 10.1038/s41598-025-95053-3

**Published:** 2025-04-28

**Authors:** Jakob J. Prömer, Sara Wolske, Perrine Castets, Geeske M. van Woerden, Cinzia Barresi, Kevin C. O’Connor, Ruth Herbst

**Affiliations:** 1 Institute for Specific Prophylaxis and Tropical Medicine, Center for Pathophysiology, Infectiology and Immunology, Medical University of Vienna, Kinderspitalgasse 15, Vienna, 1090 Austria; 2 Department of Cell Physiology and Metabolism, Faculty of Medicine, University of Geneva, 1 rue Michel Servet, Geneva, CH-1211 Switzerland; 3 Departments of Clinical Genetics, ENCORE center of expertise, Erasmus, Rotterdam, 3015 GD MC The Netherlands; 4 Neuroscience, ENCORE center of expertise, Erasmus, Rotterdam, 3015 GD MC The Netherlands; 5 Departments of Neurology, Yale School of Medicine, New Haven, Connecticut, 06511 USA; 6 Immunobiology, Yale School of Medicine, New Haven, Connecticut, 06511 USA

**Keywords:** Cell biology, Neuroscience

## Abstract

The neuromuscular junction (NMJ) is the unique interface between lower motor neurons and skeletal muscle fibers and is indispensable for muscle function. Tight control of its localized formation at the center of every muscle fiber, and maintenance throughout lifetime are sustained by muscle-specific kinase (MuSK). MuSK acts as central regulator of acetylcholine receptor clustering at the postsynapse. Localized and temporally controlled signaling of MuSK is primarily achieved by tyrosine autophosphorylation and inhibition thereof. Previous investigations suggested serine phosphorylation of the activation domain as an additional modulator of MuSK activation. Here we identified calcium/calmodulin dependent protein kinase II (CaMK2) and in particular CaMK2β as novel catalyst of MuSK activation and confirmed its capability to phosphorylate MuSK in heterologous cells. However, whereas CaMK2β absence in muscle cells reduced AChR clustering, MuSK phosphorylation was unchanged. Accordingly, we ruled out MuSK phosphorylation as the cause of synapse fragmentation in a mouse model for myotonic dystrophy type 1, in which the muscle-specific splice-variant of CaMK2β is missing, or as the cause of ataxia or delayed muscle development in CaMK2β knockout animals. Histological characterization of muscles of CaMK2β knockout mice indicated specific roles of CaMK2β in fast glycolytic versus slow oxidative muscle. Taken together, our data shows that MuSK can be phosphorylated by CaMK2β, but loss of CaMK2β is likely compensated by other CaMK2 paralogs at the NMJ.

**Supplementary Information:**

The online version contains supplementary material available at 10.1038/s41598-025-95053-3.

## Introduction

The neuromuscular junction (NMJ) is the site of contact between a motor neuron and a skeletal muscle fiber. Its formation and functional integrity are crucial for skeletal muscle contraction. The receptor tyrosine kinase (RTK) muscle-specific kinase (MuSK) together with its co-receptor low-density lipoprotein receptor-related protein 4 (Lrp4) and the motor neuron-derived ligand Agrin regulate postsynaptic differentiation including the enrichment of acetylcholine receptors (AChRs) at the NMJ^1–3^. Agrin binds Lrp4 to induce the formation of Lrp4-MuSK tetramers in which MuSK molecules are auto-phosphorylated and become enzymatically active^4^. Autophosphorylation in the juxtamembrane region relieves autoinhibition of MuSK, presumably releasing its kinase domain from a tightly locked position at the membrane and exposing a NPXY motif serving as a binding site of the intracellular adaptor protein Dok-7^5,6^. Subsequent Dok-7 binding further strengthens MuSK dimerization and facilitates a MuSK autophosphorylation activation loop, thereby boosting kinase activity to its full potential^7^. MuSK phosphorylation on at least four tyrosine residues, Y553, Y750, Y754 and Y755 is crucial for enzymatic activity and constrained by at least four inhibitory mechanisms: (1) the lack of the activating co-receptor Lrp4, (2) juxtamembrane autoinhibition, (3) lack of the activation sustaining adaptor protein Dok-7, and (4) autoinhibition of the tyrosine kinase domain by its activation loop^8^.

In previous studies, we and others have identified a novel serine phosphorylation site (S751) in the activation loop of MuSK^9,10^. S751 is phosphorylated in response to Agrin and lags tyrosine phosphorylation. Phospho-S751 is present at NMJs in adult mice. Furthermore, a phosphomimetic mutation at S751 was found to have a modulatory effect on MuSK tyrosine phosphorylation at non-saturating Agrin levels^11^. The specific function of S751 phosphorylation is unclear but current data suggest a regulatory role during MuSK activation, specifically in the presence of low levels of Agrin, which may be of biological significance in early development or ageing. Of note, other kinases also have either a serine (NTRK1/ROR1) or an amino acid with an acidic sidechain (INSR/MET) in the corresponding position of their activation domains (Supplementary Fig. S1A).

S751 is located between the critical activation loop tyrosines at positions Y750, Y754 and Y755. The sequence motif surrounding S751 does not conform to motifs of well-known serine/threonine kinases such as PKA/G/C, proline directed MAPK (ERK1/2), JNK, CK1/2 or GSK3. In addition, inhibition of MEK1/2, JNK, ERK1/2, PI3K, PKA/C/G and specific PKC subtypes did not affect S751 phosphorylation^11^.

In this study, we identified calcium/calmodulin-dependent protein kinase 2 β (CaMK2β) as the kinase phosphorylating S751 in vitro and in cells. In contrast, MuSK phosphorylation was unaffected in muscle cells and mice lacking CaMK2β. Analysis of a mouse model for myotonic dystrophy type 1 specifically lacking the muscle-specific splice variant of CaMK2β (CaMK2β_M_), which exhibits synapse fragmentation that is rescued by overexpression of CaMK2β^12–14^ revealed a global increase of MuSK protein expression but ruled out defective MuSK activation as cause of synapse fragmentation. Interestingly, global *Camk2b*-knockout in subadult mice caused changes in the fiber type proportion of slow muscle. Our results reveal MuSK as substrate for CaMK2β, and indicate that CaMK2β absence is compensated by CaMK2 paralogs.

## Results

### Kinase profiling identifies CaMK2β-dependent phosphorylation of MuSK S751

**Fig. 1:**
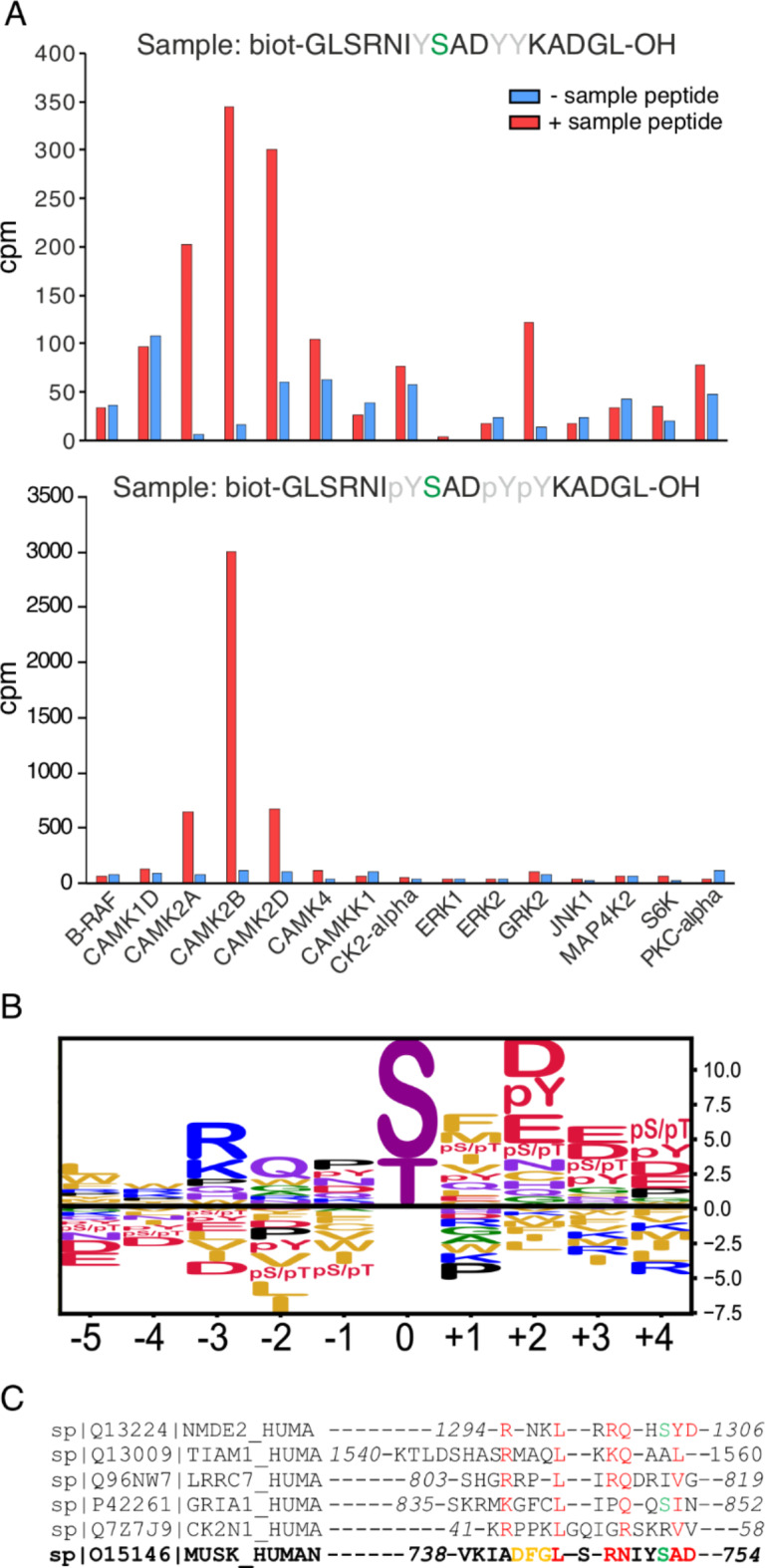
CaMK2β phosphorylates MuSK S751 *in vitro*. **A** Radiometric kinase profiling identified CaMK2α, CaMK2β and CaMK2δ as S/T kinases capable of phosphorylating a 17 amino acid peptide of the MuSK activation domain with S751 (green) located at the center. S751 phosphorylation increased strongly when three tyrosines (grey) in the peptide were phosphorylated, while activity of 242 additional S/T kinases remained basal (Supplementary Table S1). Control reactions (blue) did not contain sample peptides. **B** The substrate specificity of CaMK2 proteins matches the sequence of the MuSK activation loop peptide displayed in Fig. 1A (adapted from Jones et al. 2023). **C** The activation domain of MuSK displays sequence similarity to multiple proteins that bind to CaMK2β (adapted from Özden et al. 2024). The DFG motif (orange) is involved in ATP binding and thus not available for protein-protein interaction. Phosphorylable serine residues are marked in green, conserved residues are marked in red.

To identify kinases able to phosphorylate MuSK S751 we employed an *in vitro *kinase profiling assay covering 245 of the 360 described Ser/Thr kinases in the human kinome^15^. We used 17 AA peptides with S751 localized in the center and compared unphosphorylated peptides, and peptides with phosphorylated Y750, Y754, Y755, since tyrosine phosphorylation precedes S751 phosphorylation^9,11^. The peptides were immobilized and incubated with the respective kinase. Phosphorylation was quantified by incorporation of γ-P^33^ ATP. Within the 245 Ser/Thr kinases CaMK2α, CaMK2β and CaMK2δ were the only kinases that were able to phosphorylate non-phospho- and phospho-peptides (Fig. 1A and Supplementary Table S1). Among these three kinases, CaMK2β showed the highest activity with an 8.5-fold increase when peptides were tyrosine-phosphorylated (Fig. 1A). A recent study by Johnson and colleagues reported an atlas of substrate specificities for the S/T-kinome, which also included the identification of a specific phosphorylation-site motif for CaMK2 family proteins^15^. Consistent with the *in vitro* kinase assay data, the region surrounding MuSK S751 conforms well with the reported motif (Fig. 1B). This is further supported by recent structural evidence of CaMK2β binding to highly similar motifs in GluN2b, Tiam1, densin-180, GluA1 and CaMKIIN^16^. Of note, the activation domain of MuSK bears high sequence similarity to the peptides chosen by Özden and colleagues^16^ (Fig. 1C).

### Constitutively-active CaMK2β hyperphosphorylates MuSK S751

**Fig. 2:**
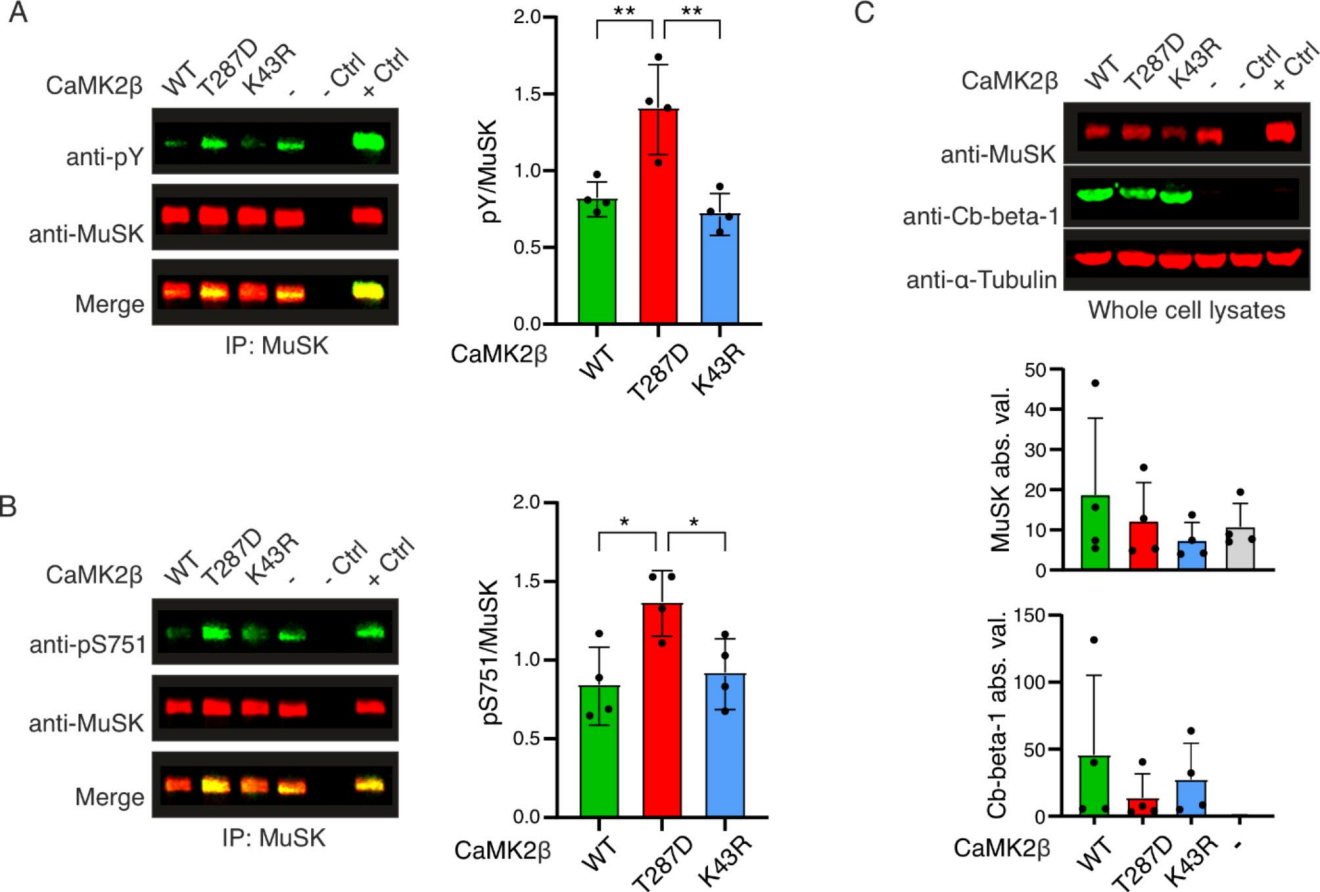
Constitutively-active CaMK2β boosts MuSK phosphorylation in heterologous cells. HEK293T cells were transiently co-transfected with MuSK and wildtype (WT), constitutively-active (T287D), or kinase-dead (K43R) CaMK2β. Co-expression of MuSK and vector plasmid (-) resulted in robust MuSK autoactivation, as expected and was used for normalization. Transfection of vector plasmid alone was used as technical negative control (- Ctrl). Transfection of constitutively-active MuSK served as technical positive control (+ Ctrl). MuSK was immunoprecipitated and subjected to immunoblotting. Membranes were probed with antibodies against tyrosine phosphorylation (pY) and MuSK (A) or activation-loop serine phosphorylation (pS751) and MuSK (B) using fluorescent immunoblotting (emission at 800 nm in green, 680 nm in red). Fluorescent signal overlaps (yellow) highlight matching band sizes. Fluorescent signal was normalized to the internal standard (-). **A** MuSK pY was increased when MuSK was co-transfected with CaMK2β T287D compared to WT (*p* = 0.0043) or K43R (*p* = 0.016). **B** MuSK pS751 was increased when MuSK was co-transfected with CaMK2β T287D compared to WT (*p* = 0.015) or K43R (*p* = 0.0421). **C** Total lysates of each experiment were subjected to immunoblotting and expression of transfected CaMK2β (Cb-beta-1) or MuSK was analyzed in absolute values (abs. val.). α-Tubulin served as loading control. Original blots are provided in Supplementary Fig. S4. All values are represented as mean ± standard deviation and were analyzed using one-way ANOVA and Tukey’s Honest Significant Difference test, *n* = 4.

 To validate the *in vitro* results, we co-expressed MuSK and CaMK2β wild-type, constitutively-active (KA) and kinase-inactive (KD) in heterologous cells. MuSK was isolated and phosphorylation was analyzed by immunoblotting. As previously described, MuSK overexpression resulted in autoactivation (Y-phosphorylation, Fig. 2A) and S751 phosphorylation (Fig. 2B), which is further increased when constitutively-active MuSK is expressed. In line with the *in vitro* kinase profiling, co-expression of CaMK2β-KA promoted a robust increase in MuSK tyrosine and serine phosphorylation (Fig. 2A, B). In contrast, co-expression of CaMK2β-wildtype or CaMK2β-KD did not increase MuSK phosphorylation beyond basal autoactivation indicating that CaMK2β-mediated MuSK phosphorylation requires full CaMK2β activation. Changes in MuSK phosphorylation were not a result of differential protein expression since expression of MuSK and CaMK2β variants were comparable (Fig. 2C). We conclude that constitutively-active CaMK2β phosphorylates MuSK S751, thereby increasing MuSK tyrosine phosphorylation and boosting kinase activity.

### Knock-out of CaMK2β in muscle cells does not affect MuSK phosphorylation but reduces AChR clustering

**Fig. 3:**
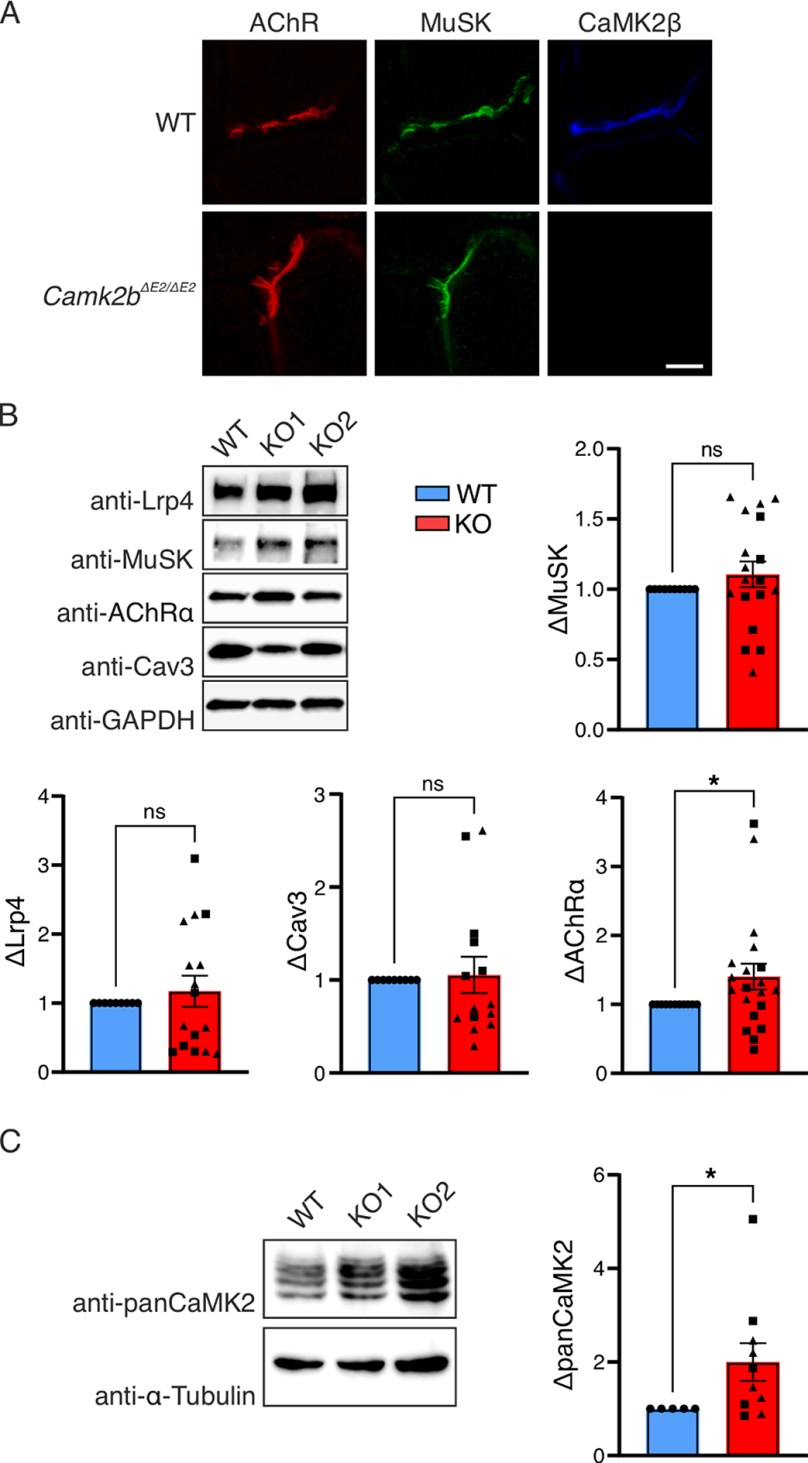
CaMK2β is localized at the NMJ and *Camk2b*-KO in muscle cells results in upregulation of AChRα and CaMK2 proteins. **A** Cryosections from *M. tibialis anterior* of WT and *Camk2b*^*-/-*^ mice were labelled with fluorescently conjugated α-BGT (red), and antibodies against MuSK (green) and CaMK2β (Cb-beta-1, blue). Confocal image stacks were acquired using a 40x oil immersion objective and displayed as maximum intensity projections, scale bar = 5 μm. Fluorescent signals co-localize at the NMJ. **B** CRISPR-Cas9 was used to generate muscle cell lines that lack all splice variants of CaMK2β by targeting exon 2 (KO1) or exon 8 (KO2) of the murine *Camk2b* gene. Total lysates were subjected to immunoblotting. Membranes were cut and probed with antibodies against proteins required for NMJ formation (Lrp4, MuSK, AChRα) or muscle cell differentiation (Cav3). Chemiluminescent signal was normalized to housekeeping protein (GAPDH). Resulting values were normalized to the WT condition to presented as change (Δ) in protein expression. *Camk2b*-KO cells displayed an increase in AChR expression (*p* = 0.0449, *n* = 11). **C** Total lysates from *Camk2b* -KO cells were assayed for expression of CaMK2 proteins (panCaMK2) using anti-panCaMK2 antibodies. *Camk2b*-KO cell lines exhibited an increase of CaMK2 proteins other than CaMK2β (*p* = 0.0346, *n* = 5). Original blots are provided in Supplementary Fig. S4. Values are represented as mean ± standard deviation and were analyzed using Welsh’s t-test, *n* ≥ 5. Dots, squares and triangles represent WT, KO1 and KO2 cell lines, respectively.

CaMK2 becomes activated when intracellular calcium levels increase, leading to calcium binding with calmodulin. The resulting calcium/calmodulin complex then binds to the autoregulatory domain of CaMK2^17^. CaMK2 is highly expressed in the brain but also present in skeletal muscle^18^. In particular, CaMK2β_M_, the muscle specific splice variant of CaMK2β is highly abundant in muscle^19–22^. Consistent with the ectopic expression experiments by Martinez-Pena and colleagues^21^, we detected CaMK2β proteins at the NMJ co-localizing with AChRs (Fig. 3A). To specifically determine the role of CaMK2β in Agrin-Lrp4-MuSK signaling, we generated muscle cells lacking CaMK2β using CRISPR/Cas9. We targeted exon 2 and exon 8 of the murine *Camk2b* gene, which are present in all *Camk2b* splice variants, to achieve a global *Camk2b* knockout (*Camk2b*-KO). Two clones derived from single cells were selected and *Camk2b*-KO was confirmed by sequence analysis and real-time RT-PCR (Supplementary Fig. 1B). There was no significant difference in expression of Lrp4 or MuSK in control or *Camk2b*-KO myotubes (Fig. 3B). We also did not observe differences in caveolin-3 (Cav3) expression, which indicates that there is no difference in cell differentiation capability (Fig. 3B). Expression of other CaMK2 variants and AChRα was increased in *Camk2b*-KO cells (Fig. 3B, C).

**Fig. 4:**
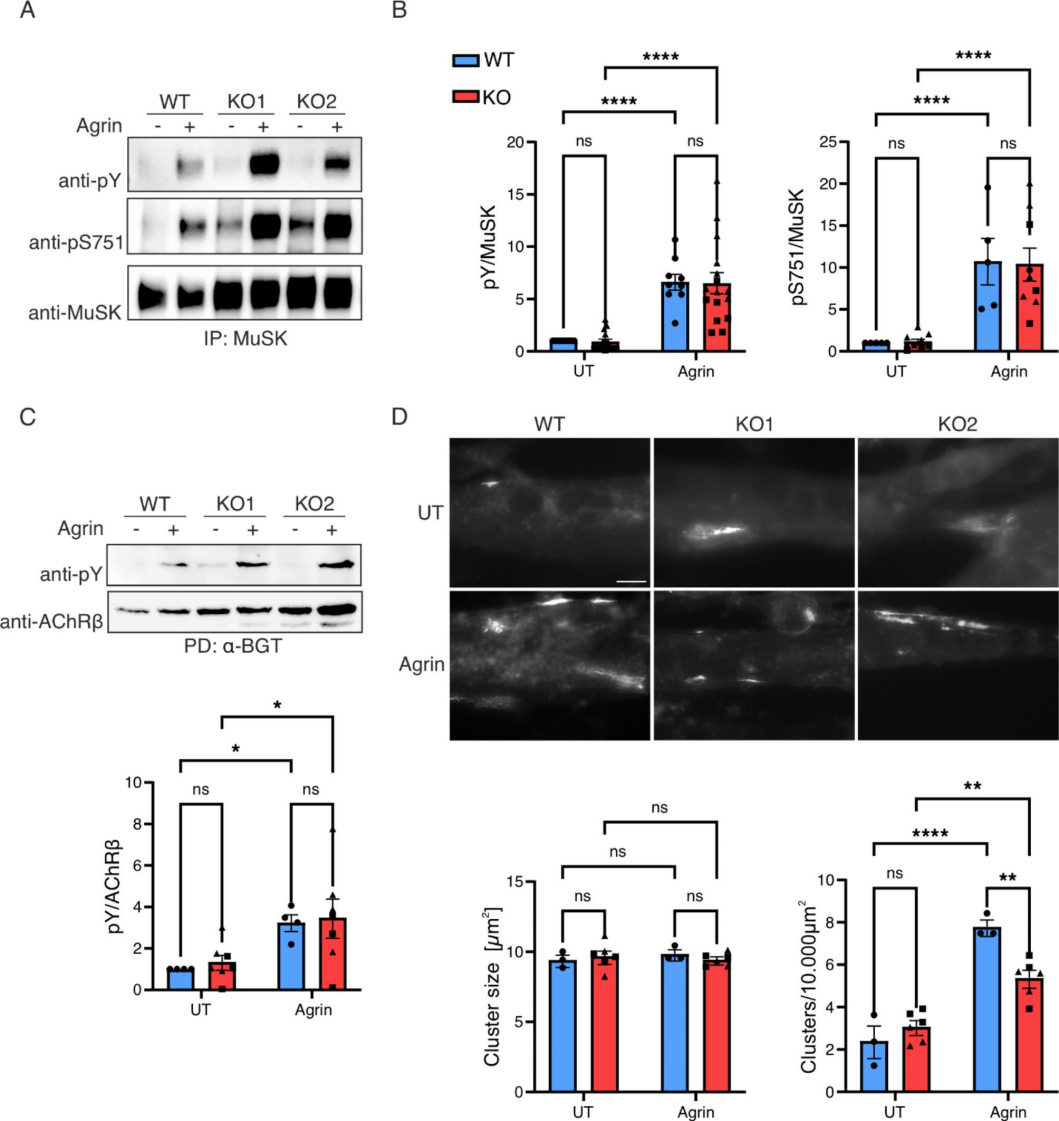
*Camk2b*-KO in muscle cells does not affect MuSK phosphorylation but reduces AChR clustering. *Camk2b*-KO was achieved by separately targeting exon 2 (KO1) or exon 8 (KO2) of the murine *Camk2b* gene in wildtype (WT) muscle cells. Differentiated muscle cells were starved for 1 h and stimulated with 0.25 nM Agrin for 1 h, as indicated. **A** MuSK tyrosine (pY) and serine 751 (pS751) phosphorylation were quantitatively analyzed from immunoprecipitated protein (IP) by chemiluminescent immunoblotting. **B** Agrin stimulation resulted in robust increase of MuSK pY in both WT and (*p* < 0.0001) and KO cells (*p* < 0.0001). Similarly, pS751 in WT (*p* < 0.0001) and KO cells (*p* < 0.0001) was increased in response to Agrin. Agrin response did not differ between WT and KO. **C** AChRs were pulled down using biotin-conjugated α-BGT. AChRβ pY was quantitatively analyzed by chemiluminescent immunoblotting. Agrin stimulation resulted in robust increase of AChRβ pY in WT (*p* = 0.0102) and KO (*p* = 0.0102) cells, *n* = 4. There was no difference in Agrin response between WT and KO cells. Original blots are provided in Supplementary Fig. S4. **D** Differentiated muscle cells were stimulated with 10 ng/ml cAgrin 4.8 for 8 h. AChR clusters were labelled using fluorescently conjugated α-BGT and documented on an inverted widefield fluorescence microscope using a 40x objective. Scale = 20 μm. Cluster size did not differ between stimulated and unstimulated, or WT and KO cells. Agrin stimulation increased the number of AChR clusters in WT (*p* < 0.0001) and KO cells (*p* = 0.0013). AChR clustering was reduced in *Camk2b*-KO compared to WT cells (*p* = 0.0046). Chemiluminescent signal, cluster number and size were normalized to the WT untreated condition. Values are represented as mean ± standard deviation. Values derived from the *Camk2b*-KO cell lines KO1 and KO2 were pooled and data were analyzed using two-way ANOVA and Šidák’s multiple comparison test, *n* ≥ 3. Dots, squares and triangles represent WT, KO1 and KO2 cell lines, respectively.

 Next, we stimulated myotubes with Agrin and isolated MuSK or AChR, and determined MuSK and AChRβ tyrosine as well as MuSK serine phosphorylation by immunoblotting. Interestingly, neither MuSK nor AChRβ phosphorylation were changed in the absence of CaMK2β (Fig. 4A-C). To interrogate whether *Camk2b*-KO cells can form AChR clusters, we induced cluster formation with Agrin and labelled AChRs with fluorescently conjugated α-BGT. We observed a significant reduction of AChR clusters in CaMK2β deficient cells, while their size was unaffected (Fig. 4D). Taken together, the lack of CaMK2β in muscle cells compromises Agrin-mediated AChR clustering, while phosphorylation of MuSK and AChRβ remain unaffected. Given that CaMK2β can phosphorylate MuSK and that calcium signaling, and therefore also CaMK2 function, differ between cultured muscle cells and intact muscle, we were prompted to examine MuSK activation in two independent mouse models lacking CaMK2β.

### MuSK phosphorylation is not affected in a model for myotonic dystrophy that specifically lacks CaMK2β_M_

The homozygous deletion of exon 3 in the splicing regulator protein muscleblind1 (*Mbnl1*^*ΔE3/ΔE3*^) results in defective splicing of the chloride channel protein 1 (*Clc-1*) and reproduces clinical manifestations of myotonic dystrophy type 1 (DM1)^12^. Patients suffer from sustained muscle contraction, which causes muscle weakness, wasting and myotonia. *Mbnl1*^*ΔE3/ΔE3*^ mice exhibit decreased expression of CaMK2β_M_ and increased expression of synaptic genes in muscle, as well as fragmented NMJs, which is rescued by overexpression of CaMK2β^13^. We reasoned that synaptic fragmentation may trace to defective interaction of CaMK2β_M_ and MuSK, and aimed to unravel the role of MuSK activation in this neuromuscular phenotype.

**Fig. 5:**
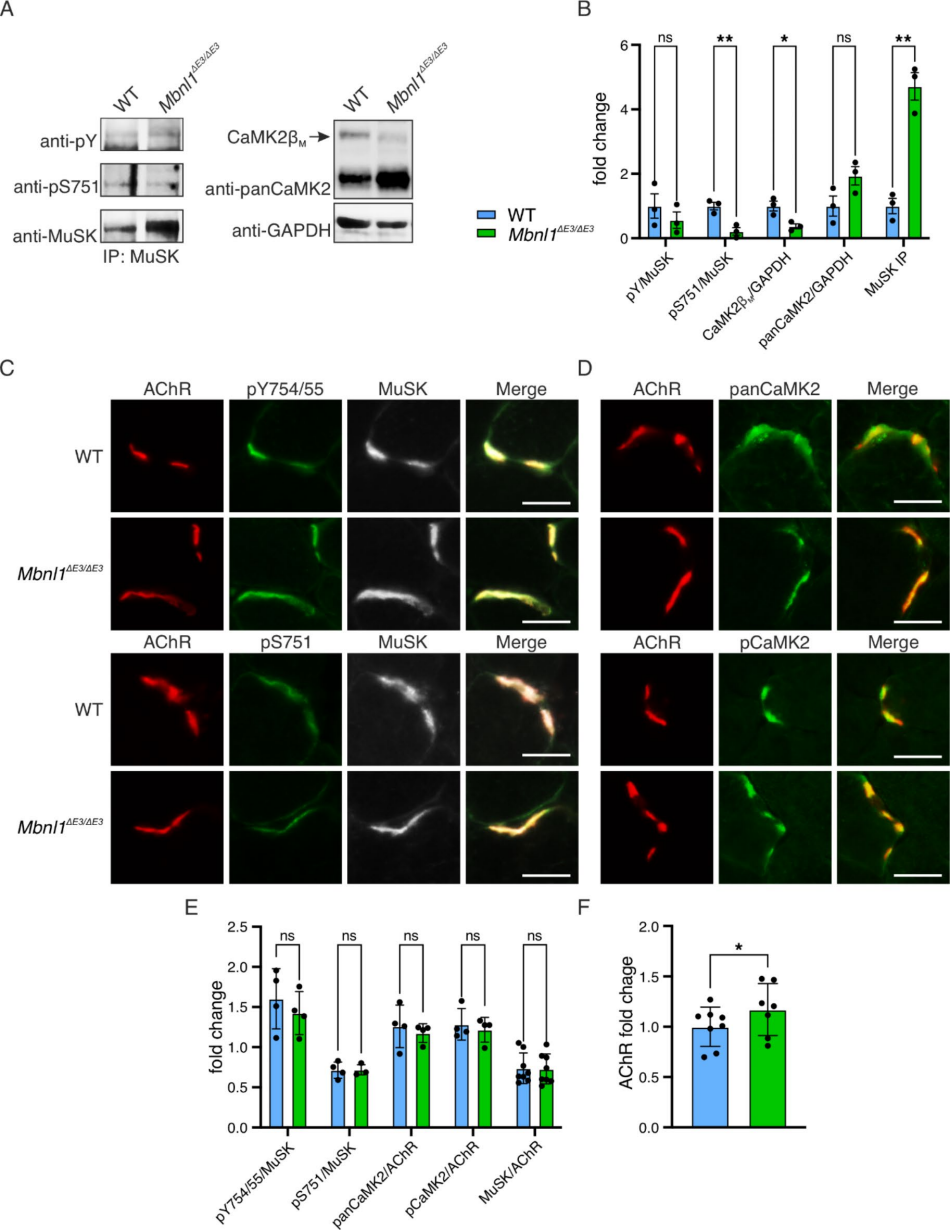
MuSK phosphorylation is not compromised at fragmented NMJs of *Mbnl1*^ΔE3/ΔE3^ mice. **A** MuSK was immunoprecipitated from *M. tibialis anterior* of WT and *Mbnl1*^ΔE3/ΔE3^ mice. MuSK pY and pS751 were quantitatively analyzed using chemiluminescent immunoblotting. Expression of CaMK2 proteins was analyzed from total lysates of corresponding animals. Original blots are provided in Supplementary Fig. S5. **B** Chemiluminescent signals were normalized as indicated. *Mbnl1*^*ΔE3/ΔE3*^ animals exhibited decreased CaMK2β_M_ protein levels as expected (Falcetta et al. 2024). Furthermore, pS751 in MuSK was reduced (*p* = 0.0090) and MuSK protein level was increased (*p* = 0.0016), Data were analyzed using unpaired two-tailed Student’s t-test, *n* = 3. **C** AChR, MuSK and its phospho-tyrosines 754/55 (pY754/55) or phospho-serine 751 (pS751) residues were labelled on 8 μm cryosections from *M. tibialis anterior* using fluorescently-conjugated α-BGT or corresponding antibodies as indicated. Images were acquired at a widefield fluorescence microscope using a 20x objective. Scale = 20 μm. **D** AChR, CaMK2β proteins (panCaMK2) or their phospho-threonine 286/7 residues (pCaMK2) were labelled on 8 μm cryosections from *M. tibialis anterior* using fluorescently-conjugated α-BGT or corresponding antibodies as indicated. Images were acquired at a widefield fluorescence microscope using a 20x objective. Scale = 20 μm. **E** At least 15 NMJs of WT or *Mbnl1*^*ΔE3/ΔE3*^ animals were manually segmented based on AChR labels. Mean fluorescent intensity of each fluorescent channel was quantified in Fiji v1.52 and normalized as indicated. Relative fluorescent intensities did not differ between WT or *Mbnl1*^ΔE3/ΔE3^. Data were analyzed using unpaired two-tailed Student’s t-test, *n* = 4. **F** Mean AChR intensity was analyzed separately to assess comparability of the groups. Values are represented as mean ± standard deviation. Data were analyzed using paired two-tailed Student’s t-test, since background differed on sample slides, *n* = 8. *Mbnl1*^*ΔE3/ΔE3*^ animals exhibited an increase in AChR levels at the NMJ (*p* = 0.0213).

 We immunoprecipitated MuSK from control and *Mbnl1*^*ΔE3/ΔE3*^ muscles and analyzed MuSK tyrosine and serine phosphorylation via immunoblotting. We observed decreased phosphorylation of MuSK in mutant muscle, while its expression was ~ 5-fold increased (Fig. 5A, B). Of note, pan-CaMK2 expression was slightly increased (*p* = 0.089) in mutant muscle (Fig. 5A, B). Next, we labelled MuSK, its phospho-residues pS751 and pY754/55, and AChRs on sections of *M. tibialis anterior* (TA) and quantitatively analyzed MuSK phosphorylation and CaMK2 accumulation at the NMJ (Fig. 5C, D). Comparison of fluorescent signal intensity revealed that MuSK phosphorylation is not altered at the NMJ in *Mbnl1*^*ΔE3/ΔE3*^ mice (Fig. 5E). We performed a similar assay labelling pan-CaMK2 proteins and their phospho-T286/7 residue. Surprisingly, there was no change in the accumulation of CaMK2 at NMJs of mutant mice (Fig. 5D, E). Fluorescent intensity of AChRs was 1.17-fold increased in *Mbnl1*^*ΔE3/ΔE3*^ mice (Fig. 5F). These results indicate that CaMK2β_M_ deficiency does not affect MuSK phosphorylation in DM1 and that other CaMK2 isoforms are upregulated in the absence of CaMK2β_M_.

### MuSK phosphorylation is unchanged in ataxic *Camk2b*^*ΔE2/ΔE2*^ mice

CaMK2β deficient mice exhibit severe motor coordination defects and delayed weight gain during development^19,23^. The cause of ataxia in these animals was linked to inverted synaptic plasticity in the parallel fiber-Purkinje cell synapse, which represents the sole output path of cerebellar signals^23^. Since our data suggested a specific interaction of CaMK2β and MuSK at the NMJ, we investigated the effect of CaMK2β deficiency on NMJ development and maintenance.

**Fig. 6:**
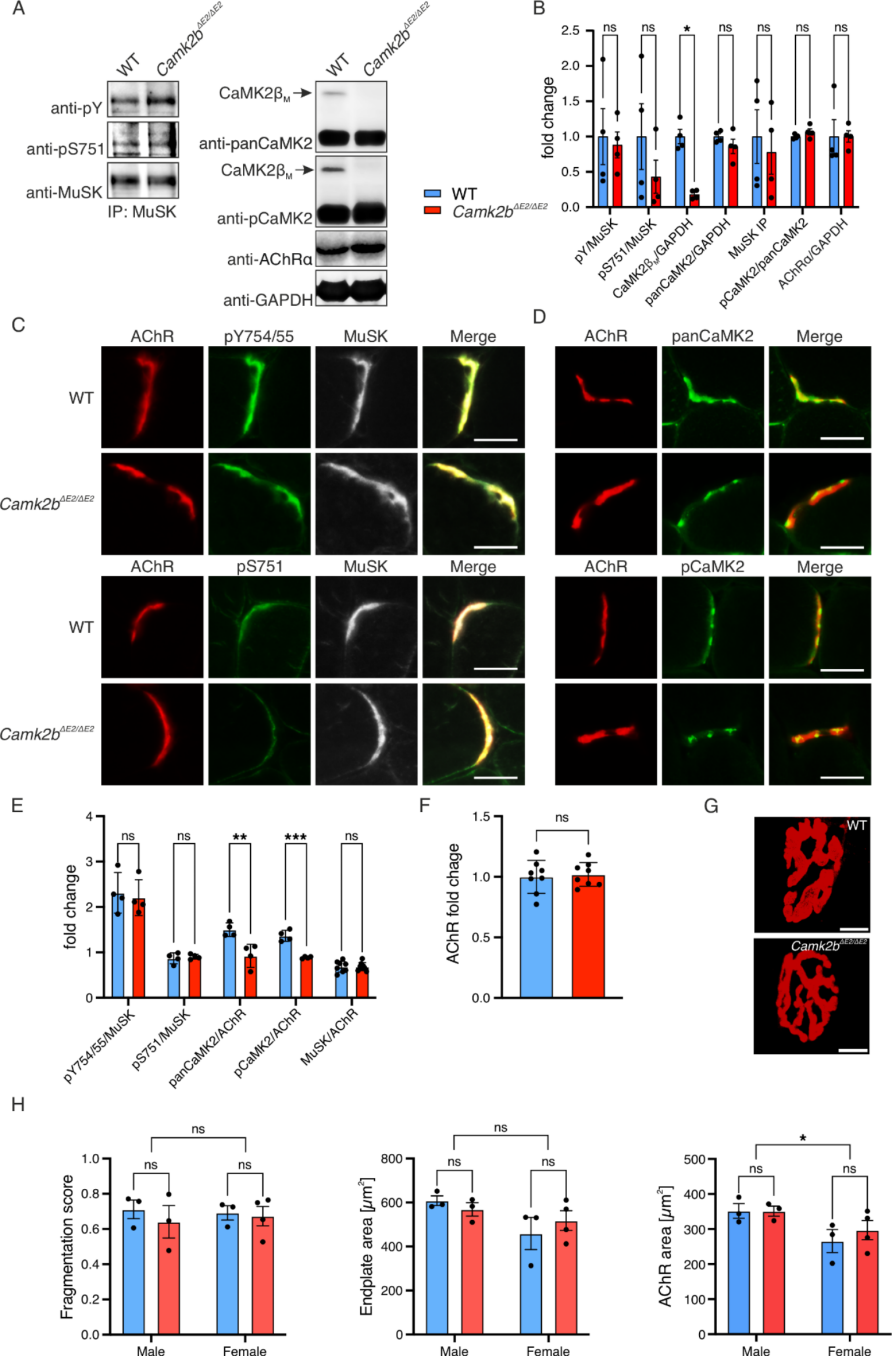
*Camk2b*^*-/-*^ mice exhibit reduced CaMK2 proteins at NMJs, yet MuSK phosphorylation and NMJ morphology are not affected. **A** MuSK was immunoprecipitated from TA of WT and *Camk2b*^*-/-*^ mice. MuSK pY and pS751 were quantitatively analyzed using chemiluminescent immunoblotting. Expression of CaMK2 proteins was analyzed from total lysates of corresponding animals. Original blots are provided in Supplementary Fig. S5. **B** Chemiluminescent signals were normalized as indicated. CaMK2β_M_ was reduced in *Camk2b*^*-/-*^ compared to WT animals (*p* = 0.0002). Data were analyzed using unpaired two-tailed Student’s t-test, *n* = 4. **C** AChR, MuSK and pY754/55 or pS751 in MuSK were labelled on 8 μm cryosections from TA using fluorescently-conjugated α-BGT or corresponding antibodies as indicated. Images were acquired at a widefield fluorescence microscope using a 20x objective. Scale = 20 μm. **D** AChR, panCaMK2 proteins or their phospho-residues (pCaMK) were labelled on 8 μm cryosections from TA using fluorescently-conjugated α-BGT or corresponding antibodies as indicated. Images were acquired at a widefield fluorescence microscope using a 20x objective. Scale = 20 μm. **E** At least 15 NMJs of WT or *Camk2b*^*-/-*^ animals were manually segmented based on AChR labels. Mean fluorescent intensity of each fluorescent channel was quantified in Fiji v1.52 and normalized as indicated. Relative fluorescence of panCamK2 (*p* = 0.0077) and phospho-CaMK2 proteins (*p* = 0.0003) was reduced in *Camk2b*^*-/-*^ animals. Data were analyzed using unpaired two-tailed Student’s t-test, *n* = 4. **F** Mean AChR intensity was analyzed separately to assess comparability of the groups. Data were analyzed using paired two-tailed Student’s t-test, since background differed on sample slides, *n* = 8. **G** NMJs from PFA-fixed diaphragm muscle were labelled using fluorescently-conjugated α-BGT. Images were acquired at a confocal laser scanning microscope using a 40x water immersion objective. Scale = 5 μm. **H** Selected variables of a standardized morphometric analysis of NMJs using a-NMJ morph (Minty et al. 2020). Male WT mice exhibited larger AChR area (*p* = 0.0198) and a tendency toward increased endplate size (*p* = 0.0641) compared to female WT mice. Endplate fragmentation was not different between the groups. Data were analyzed using two-way ANOVA and Šidák’s multiple comparison test, *n* ≥ 3). Values are represented as mean ± standard deviation.

 We isolated MuSK from control and *Camk2b*^*ΔE2/ΔE2*^ muscles and analyzed its phosphorylation via immunoblotting. As shown in Figures 6A and B, MuSK tyrosine and serine phosphorylation were unchanged in *Camk2b*^*ΔE2/ΔE2*^ muscle, as compared to controls. To identify potential compensation by other CaMK2 variants, we analyzed the expression of the additional CaMK2 proteins in total tissue lysate. We showed that CaMK2 expression as well as phosphorylation were unchanged in mutant muscle, while CaMK2β_M_ was absent, as expected (Fig. 6A, B). Next, we performed a histological analysis of MuSK phosphorylation on tibialis anterior (TA) muscle sections from control and *Camk2b*^*ΔE2/ΔE2*^ mice. We showed that MuSK phosphorylation is not altered at the NMJ in *Camk2b*^*ΔE2/ΔE2*^ mice (Fig. 6C, E). When we labelled panCaMK2 proteins and their pThr286/7 residues, we found that other CaMK2 variants accumulate at the NMJ in the absence of CaMK2β, with the total amount of CaMK2 being reduced (Fig. 6D, E). Normalized signals were comparable, as indicated by similar fluorescence intensity of AChR labels (Fig. 6F). Similarly, we observed no effect of CaMK2β depletion on MuSK phosphorylation, and a tendency toward reduction, but not absence of CaMK2 proteins at the NMJ when we conducted the same assay with *M. soleus* (soleus, Supplementary Fig. S2A, B). Interestingly, we quantified a small, but stable increase of MuSK fluorescent signal compared to AChR (Supplementary Fig. S2C). Quantification of AChR levels revealed a reduction in fluorescent intensity of labelled AChRs in *Camk2b*^*ΔE2/ΔE2*^, indicating less neurotransmitter receptor present at the NMJ (Supplementary Fig. S2D). The apparent increase in MuSK therefore results from a decrease in AChR levels at the NMJ. The alternation in AChR levels in slow, compared to fast muscle raises the notion of differential roles of CaMK2β in the respective muscle types. Taken together, a global deletion of CaMK2β does not affect MuSK activation.

**Fig. 7:**
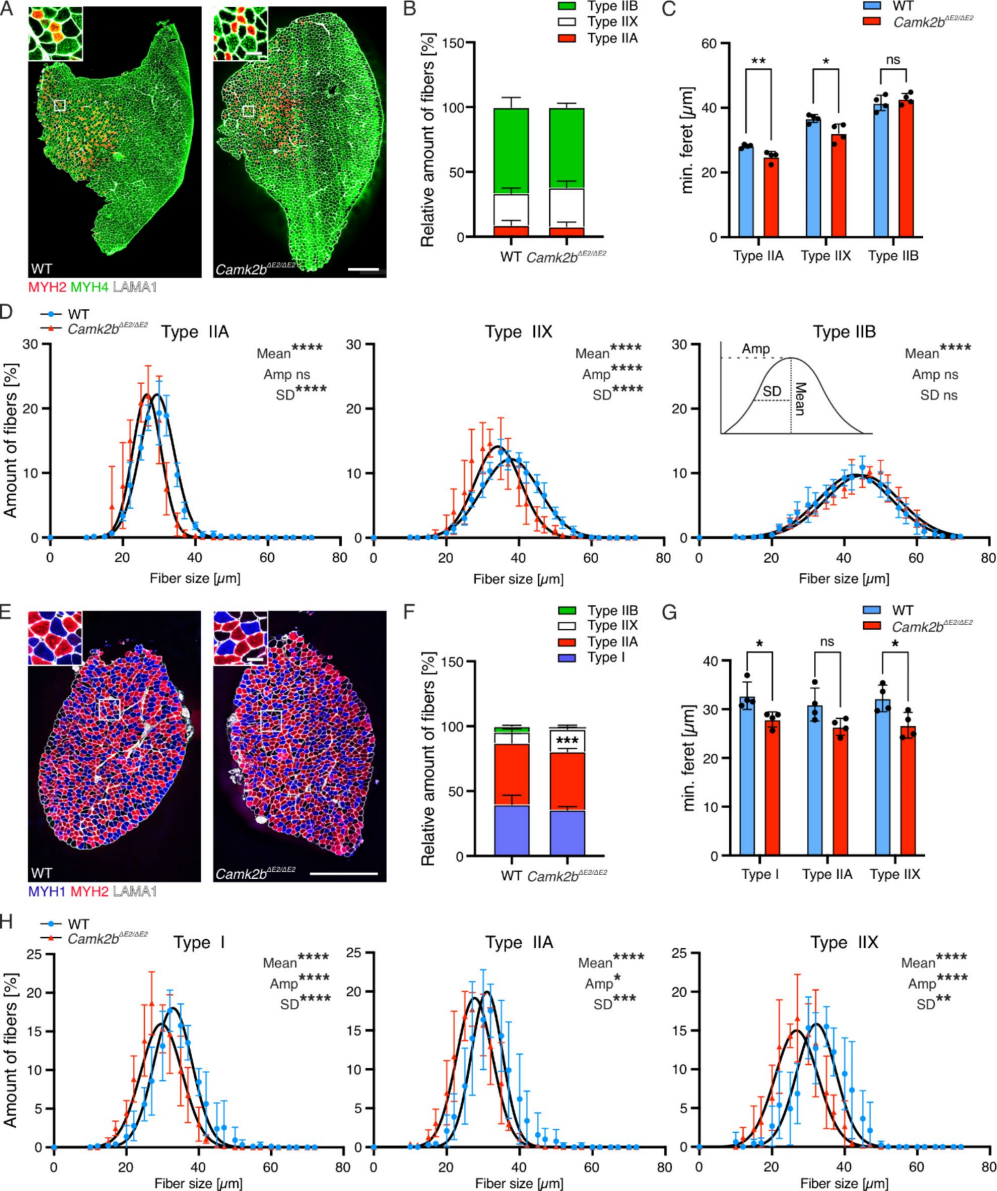
Fiber size and MyHC expression are altered in slow fibers of Camk2b^−/−^ mice. Muscle from two-month-old *Camk2b*^*−/−*^ mice was subjected to immunostaining against three isoforms of MyHC: type I (MYH1, blue), type IIA (MYH2, red) and type IIB (MYH4, green) and Laminin (LAMA1). MyHC, type IIX was determined by negative selection. Relative amounts of fiber types and size were determined according to the protocol from Ham et al. 2020. **A**,** E** Representative fluorescence images of 8 μm sections from TA (A) and soleus (E), illustrating fiber type distribution and size. Images were acquired using an upright widefield fluorescence microscope equipped with a 20x objective, and stitched. Scale overview = 500 μm, scale detail = 50 μm. **B**,** F** Relative amounts of abundant fiber types in TA (B) and soleus (F). The relative amount of type IIX fibers was higher in soleus of *Camk2b*^*−/−*^ animals (*p* = 0.0009). Data were analyzed using unpaired two-tailed Student’s t-test. **C**,** G** Quantitative analysis of type IIA and type IIX and type IIB fiber sizes in TA (C), or type I, type IIA and type IIX in soleus (G). Type IIA (*p* = 0.0097) and type IIX (*p* = 0.0252), but not type IIB fibers are smaller in TA of *Camk2b*^*−/−*^ animals. Type I (*p* = 0.0371), type IIA (*p* = 0.0688) and type IIX (*p* = 0.0304) fibers are smaller in soleus of *Camk2b*^*−/−*^ animals. Data were analyzed using unpaired two-tailed Student’s t-test. **D**,** H** Fiber size distribution of type IIA, type IIX and type IIB fibers in TA (D) or type I, type IIA and type IIX fibers in soleus (H) of WT and *CaMK2b*^*−/−*^ mice. TA: Mean size (Mean) of all Type IIA and type IIX fibers was shifted left, while mean size of type IIB fibers shifted right (*p* < 0.0001) in *CaMK2b*^*−/−*^ animals. Maximum fiber size (Amplitude, Amp) differed in type IIX fibers (*p* < 0.0001). Distribution width of type IIA and type IIX (*p* = 0.0001), but not type IIB fibers differed in *CaMK2b*^*−/−*^ animals. soleus: Mean size was shifted left in type I, type IIA and type IIX fibers (*p* < 0.0001). Maximum fiber size differed in all three fiber types (*p* < 0.0001, 0.0159, 0.0001, respectively). Size distribution width differed in all three fiber types (*p* < 0.0001, 0.0004, 0.0059, respectively). All values are represented as mean ± standard deviation. Frequency distributions were fit with gaussian distribution models and nonlinear regressions were calculated using the least squares method. Amplitude (Amp, maximum fiber size [%]), center of distribution (Mean, mean fiber size [µm]) and standard deviation (SD, width of distribution at the center of Y [µm] were compared using the extra sum of squares F test, *n* = 4.

### *Camk2b*^*ΔE2/ΔE2*^ mice develop morphologically intact NMJs and exhibit a shift in fiber type proportion in slow muscle

 To address a potential effect of CaMK2 deregulation or ataxia on NMJ integrity, we employed a morphometric analysis of NMJs in *Camk2b*^*ΔE2/ΔE2*^mice. We labelled pre- and postsynaptic densities in diaphragm muscle and primarily interrogated postsynaptic size and NMJ fragmentation. The size of the postsynapse was not affected by the lack of CaMK2β and NMJs did not show major fragmentation (Fig. 6G, H). To address previous reports of reduced body weight in subadult mice, we complemented the neuromuscular characterization of these mice with gross histological, fiber type, and fiber size analyses. We focused on TA and soleus muscles, which have different fiber type compositions and metabolic properties^24^. We found no differences in gross muscle histology using hematoxylin and eosin stains (Supplementary Fig. S3). Next, we labelled muscle sections for different myosin heavy chain (MHC) isoforms to distinguish type I, IIA, IIX and IIB fibers (Fig. 7A, B). We quantified the proportion of the respective fiber types and compared their minimum feret’s diameters (Fig. 7B, C,F, G). There was no change in fiber type distribution in TA muscle of *Camk2b*^*ΔE2/ΔE2*^ mice compared to controls (Fig. 7A, B). Interestingly, we noticed a shift from slow oxidative (I) toward fast-intermediate glycolytic fibers (IIX) in soleus from mutant mice (Fig. 7E, F). Furthermore, type IIX and IIA fibers of TA and soleus and type I fibers in soleus, but not IIB fibers in TA from *Camk2b*^*ΔE2/ΔE2*^ mice exhibited reduced diameters (Fig. 7C, D,G, H). Hence, global CaMK2β deficiency does not affect NMJ integrity in vivo, but leads to muscle atrophy in adolescent mice.

## Discussion

We have previously shown that the phosphomimetic mutation S751D stabilizes MuSK tyrosine phosphorylation upon subthreshold Agrin stimulation raising the incentive that S751 phosphorylation stabilizes MuSK kinase activity, which could be beneficial for NMJ formation and/or maintenance^11^. Our finding that CaMK2α, -β, and -δ proteins phosphorylate S751 with a strong preference for peptides carrying phosphorylated Y750, Y754 and Y755 is in line with the comprehensive substrate specificity atlas of S/T-kinases by Johnson and colleagues^15^. To our knowledge, four isoforms of CaMK2, termed β_M_, γ, δ_A_, δ_D_have been reported in striated skeletal muscle^25^, whereas CaMK2δ is the major regulator of calcium homeostasis^26^and synaptic gene expression via histone deacetylase 4 (HDAC4) in cardiac muscle^27^. Moreover, ectopic CaMK2β_M_is specifically localized at NMJs, where it participates in AChR recycling^13,21^. Thus, we focused the present study on the interaction of CaMK2β and MuSK. We validate CaMK2β localization at the NMJ, link CaMK2 to MuSK signaling, and evaluate the relevance of this novel kinase-kinase interaction for NMJ and muscle biology.

Consistent with the *in vitro* kinase assay data, we observed a coupled increase of tyrosine and S751 phosphorylation in MuSK upon co-overexpression of MuSK and kinase-active CaMK2β in heterologous cells. The fact that S751 was phosphorylated in the presence of wildtype and kinase-dead CaMK2β indicates that S751 phosphorylation does not depend on the presence of CaMK2β, but is strongly increased by kinase activation thereof. We further reason that MuSK is phosphorylated by active CaMK2β, and that kinase activation of MuSK depends on tyrosine phosphorylation, while S751 phosphorylation determines the extent of activation. These data support the hypothesis that S751 phosphorylation fosters MuSK activation^11^.

Surprisingly, knock-out of *Camk2b* in muscle cells did not abolish S751 phosphorylation. This may be due to paralog compensation, as we showed that CaMK2α -and -δ kinases also phosphorylate MuSK S751. In line with this finding, *Camk2b* KO muscle cells exhibited a robust increase in the expression of other CaMK2 entities (determined by panCaMK2 expression). A recent report also found developmental paralog compensation of *Camk2g1* in zebrafish^28^. A similar indication has emanated from the generation of viable *Camk2a*^*-/-*^ and *Camk2b*^*-/- *^mice^23,29^, while double knockout is lethal^30^. To our knowledge, CaMK2α is not present at adult NMJs. The fact that CaMK2γ did not appear in our screen leaves CaMK2δ as probable candidate to compensate for CaMK2β absence. AChR clustering was reduced in *Camk2b*-KO muscle cells, despite stable MuSK phosphorylation. It is unclear whether this effect results from defective MuSK-CaMK2β signaling or defective CaMK2β signaling alone.

CaMK2β  has important roles in synaptic organization and function in the brain^30–32^. Moreover, global knockout of CaMK2β  in mice results in reduced motor task performance and ataxia, pointing to a potential defect in neuromuscular function^19,20^. Martinez-Pena y Valenzuela and colleagues first determined a specific role of CaMK2β in AChR recycling at the NMJ^21^. Additional evidence came from the recent study of *Mbnl1*^*ΔE3/ΔE3*^ mice, which lack CaMK2β_M_ and exhibit NMJ fragmentation that was partially rescued by overexpression of CaMK2β or CaMK2β_M_^13^. These findings motivated us to analyze the *in vivo* role of CaMK2β  at the NMJ. We found that MuSK phosphorylation is decreased in muscle of *Mbnl1*^*ΔE3/ΔE3*^ mice while MuSK expression levels were elevated. Localized MuSK phosphorylation at the NMJ, however, was unchanged. This implies that *Mbnl1*^*ΔE3/ΔE3 *^mice present normal levels of active MuSK at the NMJ and increased non-phosphorylated MuSK extrasynaptically, which is in line with the report of increased extrasynaptic gene expression in this model^13^. Similar to our findings in cells, albeit less stable, we found increased CaMK2 protein levels in muscle of *Mbnl1*^*ΔE3/ΔE3*^ mice compared to wildtype. These animals exhibit increased *Camk2b *transcript levels^13^, which might explain the less pronounced upregulation of combined CaMK2 protein identities in *Mbnl1*^*ΔE3/ΔE3*^ mice compared to *Camk2b-*KO muscle cells. The fact that CaMK2 protein and activation levels (panCaMK2/AChR & pCaMK/AChR) at the NMJ were similar between wildtype and *Mbnl1*^*ΔE3/ΔE3*^ animals leaves the mechanism behind rescue of synapse fragmentation by CaMK2β overexpression at question.

In contrast to *Mbnl1*^*ΔE3/ΔE3*^ mice, *Camk2b*^*ΔE2/ΔE2*^ mice, which completely lack CaMK2β develop morphologically intact NMJs. Neither do they exhibit signs of MuSK dysregulation in biochemical analyses of whole muscle nor specifically at the NMJ. When we revisited possible isoform compensation in *Camk2b*^*ΔE2/ΔE2*^ animals, we found unchanged CaMK2 levels in tissue lysate indicating upregulation of CaMK2 isoforms other than CaMK2β, but reduced CaMK2 levels at the NMJ. This is consistent with previous reports of CaMK2β levels exceeding CaMK2γ and/or CaMK2δ levels at the NMJ, and of the latter isoforms being sufficient to guide NMJ maturation and maintenance^21^. We conclude that CaMK2β is not specifically required for NMJ maturation or maintenance and that S751 phosphorylation is well compensated, indicating high biological relevance.

CaMK2 proteins repress extrasynaptic gene expression via myogenin and HDAC4 ^33-35^. Accordingly, overexpression of CaMK2β or CaMK2β_M_ decreases *Chrna1* and *Chrng* transcription in denervated muscle of wildtype and innervated muscle of *Mbnl1*^*ΔE3/ΔE3*^ mice^13^. Increased AChRα levels in *Camk2b*-KO cells conform with this model. On the contrary, we found no indication of AChR dysregulation in *Camk2b*^*ΔE2/ΔE2*^ animals. We are currently unable to explain these differences and can only speculate that other CaMK2 variants are able to compensate in *Camk2b*^*ΔE2/ΔE2*^ mice but not in *Mbnl1*^*ΔE3/ΔE3*^ mice. In summary, we conclude that CaMK2 proteins contribute to AChR clustering and NMJ maintenance in a novel, yet unresolved mechanism that is independent of MuSK.

*Camk2b*^*ΔE2/ΔE2*^ animals exhibit ~ 15% body weight reduction compared to wildtype litter mates at two months of age^19^, the same age used herein. While no obvious pathology was visible from standard histological staining, fiber type analysis showed a slow to fast shift in slow muscle. A similar shift was found in rat muscle overexpressing CaMK2α/β^36^. This shift towards slow fibers can be explained by the ability of CaMK2 to increase myocyte enhancer factor 2 (MEF2) activity in the nucleus via HDAC4 ^37^. Elevated MEF2 activity was linked to increased slow MHC expression in skeletal muscle in mice that express constitutively-active CaMK4, which also targets HDAC4 ^38^. We therefor believe the study of specific isoform distribution and function will add information on the mechanisms and guided direction of fiber type changes. Evidence for the specific requirement of CaMK2γ and CaMK2δ in myoblast fusion, likely via phosphorylation of Rac1, a Rho GTPase required for myoblast fusion derives from a study in cultured cells^39^. The fact that weight gain in *Camk2b*^*ΔE2/ΔE2 *^mice is delayed, but not disrupted^19^ implies similar involvement of CaMK2β, and could explain generalized muscle atrophy, with the exception of type IIB fibers, which we are currently unable to explain.

Taken together, we identified a novel interaction between MuSK and CaMK2β and at the same time, excluded defective MuSK kinase regulation as a possible cause of NMJ fragmentation in *Mbnl1*^*ΔE3/ΔE3*^ mice or ataxia in *Camk2b*^*ΔE2/ΔE2*^ mice.

## Materials and methods

### Antibodies and reagents

The following primary antibodies were purchased from indicated sources: Anti-AChRα (610988/9), anti-Cav3 (610420) and anti-pY20 (610000) from Becton, Dickinson and Company (Franklin Lakes, NJ, USA), anti-AChRβ (N8283) and anti-Laminin (L9393) from Sigma-Aldrich Inc. (St. Louis, MO, USA), anti-CaMK2β (cb-beta-1) from ThermoFisher Scientific (Waltham, MA, USA), anti-CaMK2 (pan, D11A10 #4436), anti-pCaMK2 (pThr286/7, D21E4 #12716), anti-GAPDH (14C10, #2118), anti-pY100 (p-Tyr-100 #9411) and anti-α-Tubulin (DM1A #3873) from Cell Signaling Technology (Danvers, MA, USA), anti-GFP (ab290) and anti-Lrp4 (ab174637) from Abcam (Cambridge, UK), anti-MuSK (AF562) from R&D Systems Inc. (Minneapolis, MN, USA), anti-pY100 (sc-7020) from Santa Cruz Biotechnology (Dallas, TX, USA). Antibodies against MHC type I (BA-D5), type IIA (sc-71) and type IIB (10F4) were obtained from the Developmental Studies Hybridoma Bank (Iowa City, IA, USA). Primary antibody anti-MuSK IgG4 (MuSK 1 A) was expressed in HEK-293T cells using human lambda and IgG4 expression vectors respectively containing the VL and VH domains of human MuSK1A and purified as previously described^40^. Anti-pS751 was purified form 5-week immunized rabbit blood as previously described^11^. The following secondary antibodies and fluorescently-conjugated reagents were purchased from commercial sources: HRP conjugates against goat (705-035-003), mouse (115-035-003), rabbit (111-035-003) and rat (112-035-003), and fluorescent conjugates against mouse IgG1 (Cy3, 115–165-205), mouse IgG2b (Cy3, 115–165-207; 405, 115–475-207), and rabbit (Cy5, 711-605-152) from Jackson Immunoresearch (West Grove, PA, USA), anti-human (AF647, A21445) from Invitrogen (Waltham, MA, USA), anti-mouse IgM (488, A21042) from Life Technologies (Carlsbad, CA, USA). Fluorescent conjugates against goat (IR-Dye680RD, 68074; IR-Dye800CW, 32214), rabbit (IR-Dye 680RD, 68073; IR-Dye800CW, 32213), mouse (IR-Dye 680RD,68072; IR-Dye800CW, 32212) were obtained from Li-Cor Biosciences (Lincoln, NE, USA). Fluorescent conjugate of α-bugarotoxin (α-BGT, CF543, #00017) was bought from Biotium (Fremont, CA, USA). Antibody dilutions as well as additional lab reagents are available from Supplementary Table S2. Neural Agrin A4B8 was prepared from HEK 293T as reported elsewhere^5^.

### Plasmids and constructs

Oligonucleotides encoding Cas9-target sequences on exon 2 or exon 8 of the murine *Camk2b* gene were obtained from Microsynth (Balgach, Switzerland) and cloned into pX459 v2.0 (pSpCas9(BB)−2A-Puro, Addgene plasmid #62988^41^), using Bpil. Expression vectors containing MuSK wildtype (WT) and kinase-active (KA) were generated previously^11^. Human light-chain (lambda) and heavy-chain (IgG4) expression vectors containing the VL and VH domains of human anti-MuSK (MuSK 1 A) were previously described^40^. Rat CaMK2β-WT and constitutively-active (KA, T287D) were cloned in the laboratory of Geeske van Woerden into a dual promoter expression vector as described in Küry, et al.^42^. CaMK2β kinase-dead (KD, K43R) was generated by site-directed mutagenesis using the primers FW-TATGCAGCTAgGATCATTAACACCAAGAAG and RV-CTCATGGCCGGTGCAGAG.

### Radiometric protein kinase assay/^33^PanQuinase activity assay

The phosphorylation profile of biotinylated 17 amino acid peptides that resemble the activation domain of MuSK were determined on a panel of 245 S/T-kinases by ProQinase (Reaction Biology, Freiburg, Germany) (Supplementary Table S1). Peptides were synthesized in triply-tyrosine phosphorylated (biot-GLSRNI(p)YSAD(p)Y(p)YKADGL-OH) and unphosphorylated states, and provided at 200 mM concentration in 300 µl 50 mM Hepes, pH 7.5 with 1.5% (v/v) or without DMSO, respectively (Intavis peptide services, Tübingen, Germany). Peptides were used at 1 µM final concentration with 1–400 ng sample kinase in 50 µl reactions. The reaction buffer contained 50 mM Hepes-NaOH, pH 7.5 supplemented with 3 mM MgCl_2_, 3 mM MnCl_2_, 3 µM Na_3_O_4_V, 1.2 mM DDT, 1 µM ATP/[γ-^33^P]-ATP. PKC assays except the PKC-mu and the PKC-nu assay additionally contained 1 mM CaCl_2_, 4 mM EDTA, 5 µg/ml Phosphatidylserine and 1 µg/ml 1.2-Dioleyl-glycerol. The MYLK2, CaMK1δ, CaMK2α, CaMK2β, CaMK2δ, CaMK4, CaMKK2 and DAPK2 assays additionally contained 1 µg/ml Calmodulin and 0.5 mM CaCl_2_. The PRKG1 and PRKG2 assays additionally contained 1 µM cGMP. Reaction mixtures were incubated at 30 °C for 60 min and stopped from reacting by addition of 20 µl of 4.7 M NaCl/35 mM EDTA. Reaction mixtures were transferred to streptavidin-coated flash-plates and incubated at room temperature for 30 min. After washing with 150 mM NaCl, incorporation of [γ-^33^P]-ATP was determined using a microplate scintillation counter (Microbeta, Perkin Elmer, Waltham, MA, USA). Background signals for each individual kinase were determined in parallel.

### Cell culture

HEK 293T cells were purchased from ATCC (Manassas, VA, USA) and cultured at 37 °C and 5% CO_2_ in Dulbecco’s Modified Eagle’s Medium high glucose (DMEM, Sigma-Aldrich) supplemented with 10% fetal bovine serum (FBS) and 1% (v/v) penicillin/streptomycin (Sigma-Aldrich). For experimental procedures, cells were plated to reach 80% confluency the following day and transfected using 2 µg plasmid DNA (1 µg MuSK + 1 µg CaMK2β or pCDNA 3.1, the latter vector was used as empty control) and 10 µl linear PEI (pH 7, Polysciences Inc, Warrington, PA, USA). Cells were lysed 48 h after transfection and subjected to biochemical preparation and immunoblotting.

Muscle cells previously generated from C57/BL6 embryos carry a temperature-sensitive SV40 T antigen and were propagated at 33 °C at 5% CO_2_. They were cultured in growth medium composed of DMEM containing glutamine, 4.5 g/l glucose enriched with 10% (v/v) FBS, 10% (v/v) horse serum (HS), 20 U/ml recombinant murine interferon-γ (IFN-γ; Peprotech) and 1% (v/v) penicillin/streptomycin^5^. To obtain myotubes, cells were seeded at ~ 80% confluency and kept at 33 °C for one night. The following day, medium was changed to differentiation medium, composed of DMEM supplemented with 10% HS and 1% (v/v) penicillin/streptomycin and refreshed every 2–3 days. Cells were subjected to experimental procedures 4–5 days after initiation of differentiation. To determine MuSK or AChR phosphorylation, differentiated myotubes were starved 1 h with DMEM and stimulated with 0.25 nM neural Agrin A4B8 ^43^ for 60 min, before being subjected to biochemical preparation.

### Generation of CaMK2β-deficient muscle cells

Muscle cells deficient of CaMK2β were generated by transient transfection of pSpCas9(BB)−2A-Puro (PX459) v2.0 (Addgene plasmid #62988^41^). We targeted exon 2 or exon 8 of the murine *Camk2b* gene (ensembl: ENSMUSG00000057897). Transfected cells were selected using 3 µg/ml puromycin for two consecutive days. Remaining were cells isolated and expanded as separate clonal cell lines. Individual cell lines were assayed for their capability to differentiate and the expression of NMJ related proteins. Absence of CaMK2β was examined by sequence analysis of PCR-amplified genomic DNA, and quantitative RT-PCR of individual cell lines using locus spanning primers. Two clones—in which either exon 2 (termed KO1) or exon 8 (termed KO2) was targeted—fulfilled these requirements and were chosen for subsequent experimentation. Primers and oligonucleotides are available from Supplementary Table S2.

### Animals

*Mbnl1*-deficient mice (*Mbnl1*^*ΔE3/ΔE3*^) animals were obtained from Swanson et al.^12^. *Camk2b* deficient mice (*Camk2b*^*ΔE2/ΔE2*^) were generated previously^20^. Two months old *Camk2b*^*ΔE2/ΔE2*^ mice were euthanized using carbon dioxide, three months old *Mbnl1* mice were euthanized using a lethal dose of pentobarbital, and tissues were dissected. Female *Camk2b*^*ΔE2/ΔE2*^ mice were used for morphometric analysis of NMJs. Both sexes of *Mbnl1* mice were considered for biochemical and histological analyses. All experiments were conducted in accordance with the European Commission Council Directive 2010/63/EU and all described experiments and protocols were ethically approved by an independent review board of the Erasmus MC (CCD project license AVD101002017893) and the Veterinary Office of the Canton of Geneva (application number GE220/GE227). Animal husbandry and experimentation were performed according to the ARRIVE (Animal Research: Reporting of In Vivo Experiments) guidelines.

### Biochemical Preparation and Immunoblotting of cell and tissue samples

Cells were resuspended in RIPA buffer (50 mM Tris pH 7.5, 150 mM NaCl, 1 mM EDTA, 1% NP-40, 0.1% SDS, 0.5% sodium deoxycholate) supplemented with protease inhibitors (1 µg/ml aprotinin, 1 µg/ml leupeptin, 1 µg/ml pepstatin, 20 µM PMSF) and phosphatase inhibitor (1 mM Na_3_O_4_V). Cell lysates were cleared at 16.000 g for 15 min. Protein concentration of cell lysates was determined using a PCA-enhanced biuret reaction kit (RotiQuant Universal, Karlsruhe, Germany). Total cell lysates were subjected to SDS-PAGE and immunoblotting contained 10 µg of protein. MuSK was immunoprecipitated from 0.1 mg total protein lysate of HEK 293T cells or 0.4 mg total protein of muscle cell cultures. Lysates were incubated overnight with anti-MuSK MuSK1A mAb and protein G agarose (Roche Diagnostics). AChRs were isolated by incubating 0.2 mg muscle cell lysate with biotin-conjugated α-bungarotoxin (α-BGT) for 2 h and subsequent incubation of lysates with streptavidin-conjugated agarose resin (ThermoFisher, Waltham, MA, USA). Proteins were transferred to nitrocellulose membranes (Cytiva, Malborough, MA, USA) using a semi-dry transfer blotter (Bio-Rad). To detect several proteins simultaneously, and to minimize interference of stripping, membranes were cut prior to antibody hybridization as indicated in Supplementary Figures S4-6. In the case of MuSK-IPs, membranes were cut to remove bands corresponding to IgGs. Stripping and re-probing are indicated where applicable in Supplementary Figures S4-6. MuSK phosphorylation in heterologous cells was analyzed using near-infrared fluorescence immunoblotting. Biochemical analyses of muscle cells were performed using Immobilon chemiluminescent HRP substrate (Merck, Burlington, MA, USA).

Tissue samples were minced after dissection (*Mbnl1*^*ΔE3/ΔE3*^ TA) or snap frozen in liquid N_2_ and crushed (*Camk2b*^*ΔE2/ΔE2*^ TA), and processed with a Dounce homogenizer. Tissue lysates were cleared at 20.000 g for 30 min. Protein concentration of tissue lysates was determined using a commercially available BCA-kit (ThermoFisher, Waltham, MA, USA). Tissue lysates subjected to SDS-PAGE and immunoblotting contained 25 µg of protein. MuSK was immunoprecipitated from 2 mg TA protein lysate of *Camk2b*^*ΔE2/ΔE2*^ animals or 5 mg of *M. gastrocnemius* protein lysate of *Mbnl1*^*ΔE3/ΔE3*^ animals as described for cell lysates. Proteins were transferred to nitrocellulose membranes using semi-dry (*Camk2b*^*ΔE2/ΔE2*^) or tank transfer methods (*Mbnl1*^*ΔE3/ΔE3*^). Biochemical analyses of tissue were performed using chemiluminescence revelation techniques.

All membranes were probed with antibodies as indicated in the respective figures at dilutions available from Supplementary Table S2. Signals were detected on a Li-Cor Odyssey XF imaging system and analyzed using Empiria Studio version 3.0.0.173 (Li-Cor Biosciences, Lincoln, NE, USA). Uncropped blots are shown in Supplementary Figures S4 −6. All raw data and quantification files of immunoblot experiments are available as a publication pack from Zenodo.org (https://zenodo.org/records/14881200).

### AChR clustering and NMJ morphometry

To assay AChR clustering, differentiated muscle cells were stimulated with 10 ng/ml neural Agrin A4B8 for 8 h. Cells were fixed with 2% paraformaldehyde (PFA) and AChR clusters were labelled with CF543-conjugated α-BGT (Biotium, Fremont, CA, USA) and mounted using Mowiol 4–88. Clusters were documented using a Zeiss Axiovert 200 using a 40x oil immersion objective (Zeiss, Oberkochen, Germany) and Metamorph software (Molecular Devices, Sunnyvale, USA). At least 30 images per sample were analyzed using a previously published macro^44^ in Fiji version 1.52v^45^.

*Camk2b*^*ΔE2/ΔE2*^ mice were perfused with 4% PFA in PBS and diaphragmatic muscle was dissected. Right hemidiaphragms from female animals were subject to immunohistochemical preparation. Tissue was pretreated with 0.1 M glycine in PBS (pH 7.3), blocked 1 h at room temperature in 5% FBS in PBS plus 0.5% Triton X-100 and incubated with primary antibodies blocking solution at indicated dilutions (Supplementary Table S2) for 20 h. Tissues were thoroughly washed by flushing them with PBS multiple times over three hours, and incubated with secondary antibodies overnight. Tissues were mounted in Vectashield (H-1000-10, Vector Labs, Newark, CA, USA) after another cycle of thorough washing over three hours. NMJs were imaged en-face on a Zeiss LSM-700 confocal laser scanning microscope using a 40x water immersion objective (Zeiss, Oberkochen, Germany). Maximum intensity projections of imaging stacks were analyzed using aNMJ-Morph, a standardized image analysis tool intended for NMJ morphometry in Fiji v1.52 ^46^.

### Immunohistological analysis of muscle sections

MuSK phosphorylation and CaMK2 expression at the NMJ were assayed using cryopreserved tissue samples. TA from *Camk2b*^*ΔE2/ΔE2*^ or *Mbnl1*^*ΔE3/ΔE3*^; and soleus from *Camk2b*^*ΔE2/ΔE2*^ mice were frozen using liquid-N_2_ cooled isopentane. AChRs, MuSK, and specific phospho-residues in MuSK were labelled on 8 μm thick cryosections. Fluorescent signals were documented on a Zeiss AxioCam Fluo microscope using a 20x air objective (Zeiss, Oberkochen, Germany). NMJs were manually segmented in Fiji version 1.52v using α-BGT as reference signal. Mean intensity of α-BGT, MuSK and phospho-MuSK was quantified in the same segmented regions of interest. Data were screened for homogeneity in Microsoft Excel version 16.87 (Microsoft, Redmond, WA, USA). Phospho-signal was normalized to MuSK signal. MuSK, pan-CaMK2 or pCaMK2 protein signals were normalized to AChR signals.

To localize pan-CaMK2 or CaMK2β proteins at the NMJ, we used antibodies detecting the majority of CaMK2 proteins (panCaMK), or an antibody specific for CaMK2β proteins (Cb-beta1). The “mouse on mouse” kit from Vectorlabs (BMK-2202) was used on cryosections of TA of wildtype and *Camk2b*^*ΔE2/ΔE2*^ mice. 8 μm sections were imaged as 1 μm optical slices on a Zeiss LSM-700 confocal laser scanning microscope using a 40x oil immersion objective (Cb-beta1) and analyzed as maximum intensity projections.

To achieve a fiber type characterization of TA and soleus of *Camk2b*^*ΔE2/ΔE2*^ mice, 8 μm cryosections of each muscle were labelled with antibodies against LAMA1, MYH1, MYH2 and MYH4. Fibers predominantly expressing MHC type IIX were determined by negative selection. Laminin labelling was used to determine minimum feret’s diameters of each fiber. Fiber type and size were quantitatively analyzed following the protocol by Ham, et al.^47^.

### Quantification and statistical analysis

GraphPad Prism version 10.1.1 (Boston, Massachusetts USA, www.graphpad.com) was used for data visualization and statistical analyses. Statistical tests and comparisons are indicated in respective figure legends. The threshold for statistical significance was set to *p* < 0.05.

## Electronic supplementary material

Below is the link to the electronic supplementary material.

Supplementary Material 1

Supplementary Material 2

## Data Availability

The raw data supporting the conclusions of this article have been deposited at https://zenodo.org/records/14881200 and will be made available by the authors (J.J.P and R.H.) upon request.
