# Supplementary material for: MuSK is a substrate for CaMK2β but this interaction is dispensable for MuSK activation in vivo
Source: Sci Rep. 2025 Apr 28;15:14865. doi: 10.1038/s41598-025-95053-3 (PMC12037915; doi:10.1038/s41598-025-95053-3)
Supplement: Supplementary file 2 — Supplementary Material 2 [file 41598_2025_95053_MOESM2_ESM.pdf]

Supplementary Information

MuSK is a substrate for CaMK2β but this interaction is dispensable for MuSK activation *in vivo*

Jakob J. Prömer[1], Sara Wolske[1], Perrine Castets[2], Geeske M. van Woerden[3,4], Cinzia Barresi[1], Kevin C. O'Connor[5,6], Ruth Herbst[1]

Supplementary Table S2. Information on antibodies and other reagents

**primary antibodies**	**IB**	**IP**	**IHC**	**Vendor/Source**	**Cat#**
anti-AChRα	1:500			BD Transduction Laboratories	610988/-9
anti-AChRβ	1:3000			Sigma-Aldrich, Inc.	N8283
anti-CaMKII beta	1:15000		1:100	Thermo Fisher Scientific	CB-beta-1
anti-CaMKII (pan)	1:1000		1:100	Cell Signaling Technology	D11A10 #4436
anti-CaMKII (pThr 287)	1:1000		1:250	Cell Signaling Technology	D21E4 #12716
anti-Cav3	1:1000			BD Transduction Laboratories	610420
anti-GAPDH	1:1000			Cell Signaling Technology	14C10 #2118
anti-GFP	1:2500			Abcam	ab290
anti-Laminin	1:200			Sigma-Aldrich, Inc.	L9393
anti-Lrp4	1:2000			Abcam	ab174637
anti-MHC type I			1:100	Developmental Studies Hybridoma Bank	BA-D5
anti-MHC type IIa			1:200	Developmental Studies Hybridoma Bank	sc-71
anti-MHC type IIb			1:100	Developmental Studies Hybridoma Bank	10F5
anti-MuSK	1:1000			R&D Systems Inc.	AF562
anti-MuSK 189-1		1:500	1:250	Takata et al. 2019	
anti-pS751	1:5000		1:300	Camurdanoglu et al. 2016	
anti-pY20	1:1000			BD Transduction Laboratories	610000
anti-pY99	1:1000			Santa Cruz Biotechnology	sc-7020
anti-pY100	1:2000			Cell Signaling Technology	p-Tyr-100 #9411
anti-Tubα	1:5000			Cell Signaling Technology	DM1A #3873
**Secondary antibodies**					
anti-goat HRP	1:10000			Jackson Immunoresearch	705-035-003
anti-human AF647			1:1000	invitrogen	A21445
anti-mouse HRP	1:10000			Jackson Immunoresearch	115-035-003
anti-mouse IgG1 Cy3			1:500	Jackson Immunoresearch	115-165-205
anti-mouse IgG2b Cy3			1:500	Jackson Immunoresearch	115-165-207
anti-mouse IgM 488			1:250	Life Technologies	A21042
anti-mouse IgG2b 405			1:250	Jackson Immunoresearch	115-475-207
anti-rabbit 647			1:500	Jackson Immunoresearch	711-605-152
anti-rabbit HRP	1:10000			Jackson Immunoresearch	111-035-003
anti-rat HRP	1:10000			Jackson Immunoresearch	112-035-003
IR-Dye 680RD donkey anti goat	1:20000			Li-Cor Biosciences	68074
IR-Dye 680RD donkey anti rabbit	1:20000			Li-Cor Biosciences	68073
IR-Dye 680RD donkey anti mouse	1:20000			Li-Cor Biosciences	68072

IR-Dye 800CW donkey anti goat	1:20000			Li-Cor Biosciences	32214
IR-Dye 800CW donkey anti rabbit	1:20000			Li-Cor Biosciences	32213
IR-Dye 800CW donkey anti mouse	1:20000			Li-Cor Biosciences	32212
**Reagents**					
α-Bungarotoxin, AF555			1:500	Invitrogen	B35451
biotin-α-Bungarotoxin		1:5000		Biotium, Inc.	#00017
protein G agarose				Roche	11719416001
Clarity Westren ECL substrate				Bio-Rad	#1705061
Immobilon Western HRP substrate				Merck Millipore	WBKLS0500
linear Polyethylenimine (PEI)				Polysciences Inc.	23966-100
Vectashield				Vector Labs	H-1000-10
Vectashield with DAPI				Vector Labs	H-1200-10
**CaMK2β KO cell line generation**					
guide RNAs					
Exon 2	Forward	caccgTACAGAGCTTGACACAGCGT			
	Reverse	aaaCACGCTGTGTCAAGCTCTGTAC			
Exon 8	Forward	caccGATGTCCACAGGTTTGCCGT			
	Reverse	aaaCACGGCAAACCTGTGGACATC			
Exon spanning primers	Forward	ACCTTCCATGAGTCCCTATGTCCAGC			
Exon 2	Reverse	CCTGCAGGCTTGGGAAGGAGAAGA			
	Forward	GCAGAGGGTACTGGCAGCTCCT			
Exon 8	Reverse	GTCCTCAGTGTCCCTTTGCGCC			

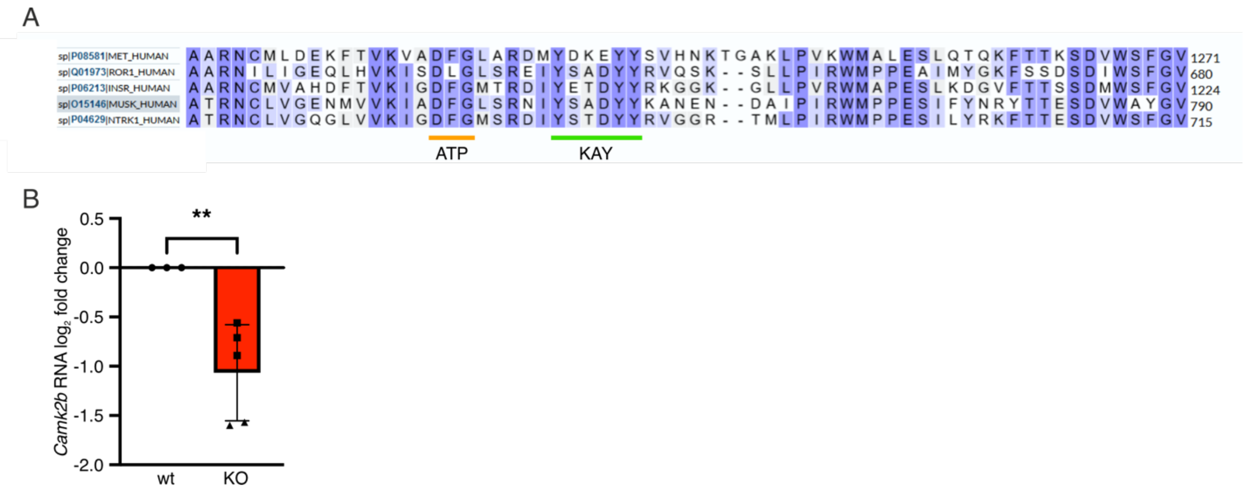

Supplementary Figure S1. Sequence alignment of MuSK with four RTKs and qRT-PCR of *Camk2b*-KO muscle cells. A ATP-binding region (ATP) and kinase activating tyrosines (KAY) are highlighted in orange and green, respectively. **B** Muscle cells were transiently transfected with Cas9 and gRNAs to target exon 2 or exon 8 the *Camk2b*-gene (ENSMUSG00000057897) and achieve a *Camk2b*-Knockout (KO). Single cell clones were isolated and two KO cell lines were selected. KO-cell lines were differentiated into myotubes, RNA was isolated and subjected to quantitative RT-PCR. KO cells exhibited a robust reduction of *Camk2b* transcript ($p = 0.0081$). Values are represented as mean ± standard deviation and data were analyzed using Welch's t-test, $n = 3$. Dots, squares and triangles represent WT, KO1 and KO2 cell lines, respectively.

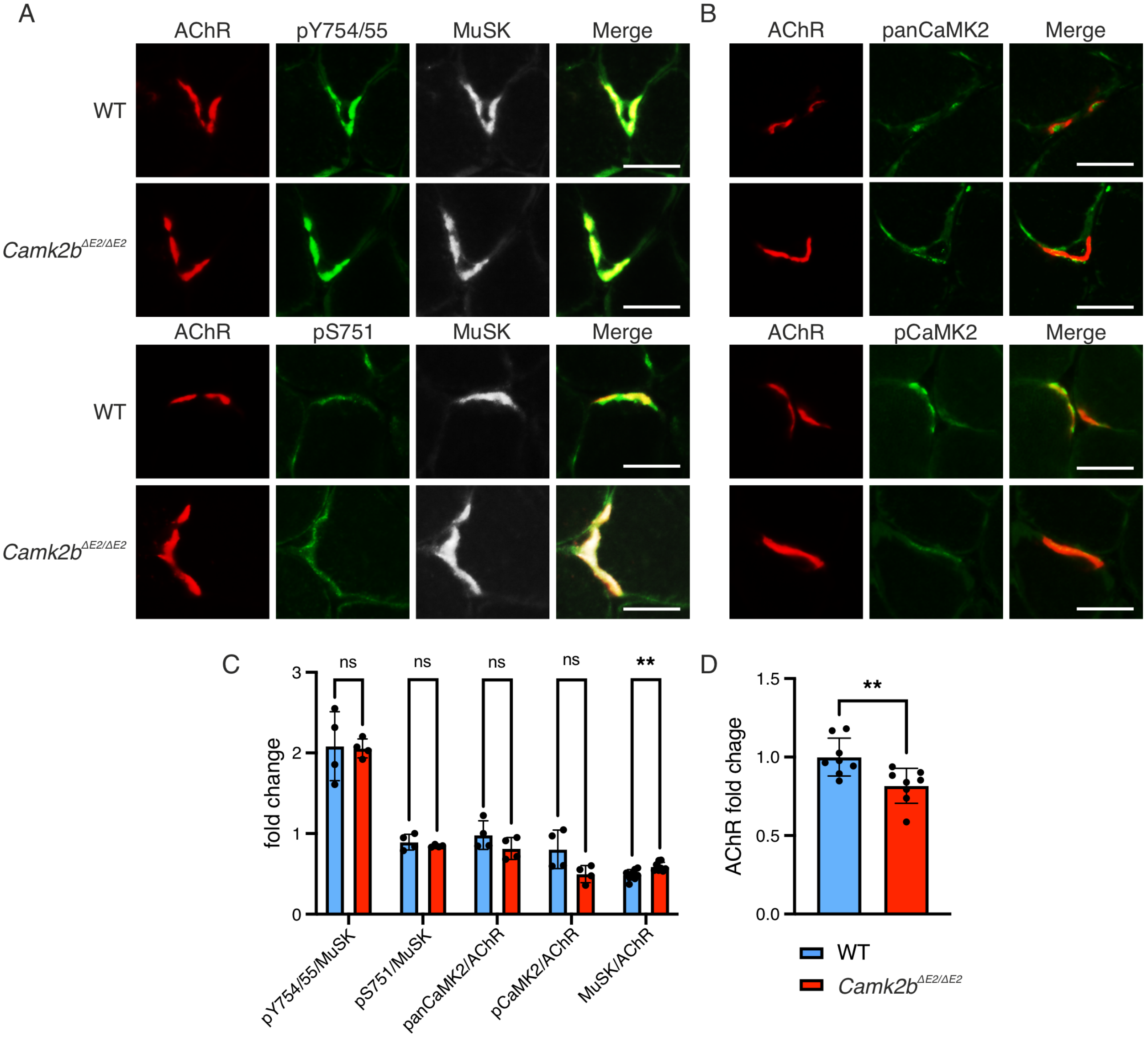

Supplementary Figure S2. *Camk2b$^{-/-}$* exhibit a reduction of AChR proteins at the NMJ in *M. soleus*. A AChR, MuSK and pY754/55 or pS751 in MuSK were labelled on 8 µm cryosections from *M. soleus* using fluorescently conjugated α-BGT or corresponding antibodies as indicated. Images were acquired at a widefield fluorescence microscope using a 20x objective. Scale = 20 µm. **B** AChR, panCaMK2 proteins or their phospho-residues (pCaMK2) were labelled on 8 µm cryosections from *M. soleus* using fluorescently conjugated α-BGT or corresponding antibodies as indicated. Images were acquired at a widefield fluorescence microscope using a 20x objective. Scale = 20 µm. **C** At least 7 NMJs of WT or *Camk2b$^{-/-}$* animals were manually segmented based on AChR labels. Mean fluorescent intensity of each fluorescent channel was quantified in Fiji v1.52 and normalized as indicated. Relative fluorescence of pCaMK2 was reduced in *Camk2b$^{-/-}$* animals ($p = 0.0569$). Relative MuSK fluorescence was increased at NMJs of *Camk2b$^{-/-}$* animals

(p = 0.0050). Values are represented as mean ± standard deviation. Data were analyzed using unpaired two-tailed Student's t-test, n = 4.**D** Mean AChR intensity was analyzed separately to assess comparability of the groups. Data were analyzed using paired two-tailed Student's t-test, since background differed between samples, n = 8. *Camk2b*$^{-/-}$ animals exhibited less fluorescent intensity of AChR labels (p = 0.0129).

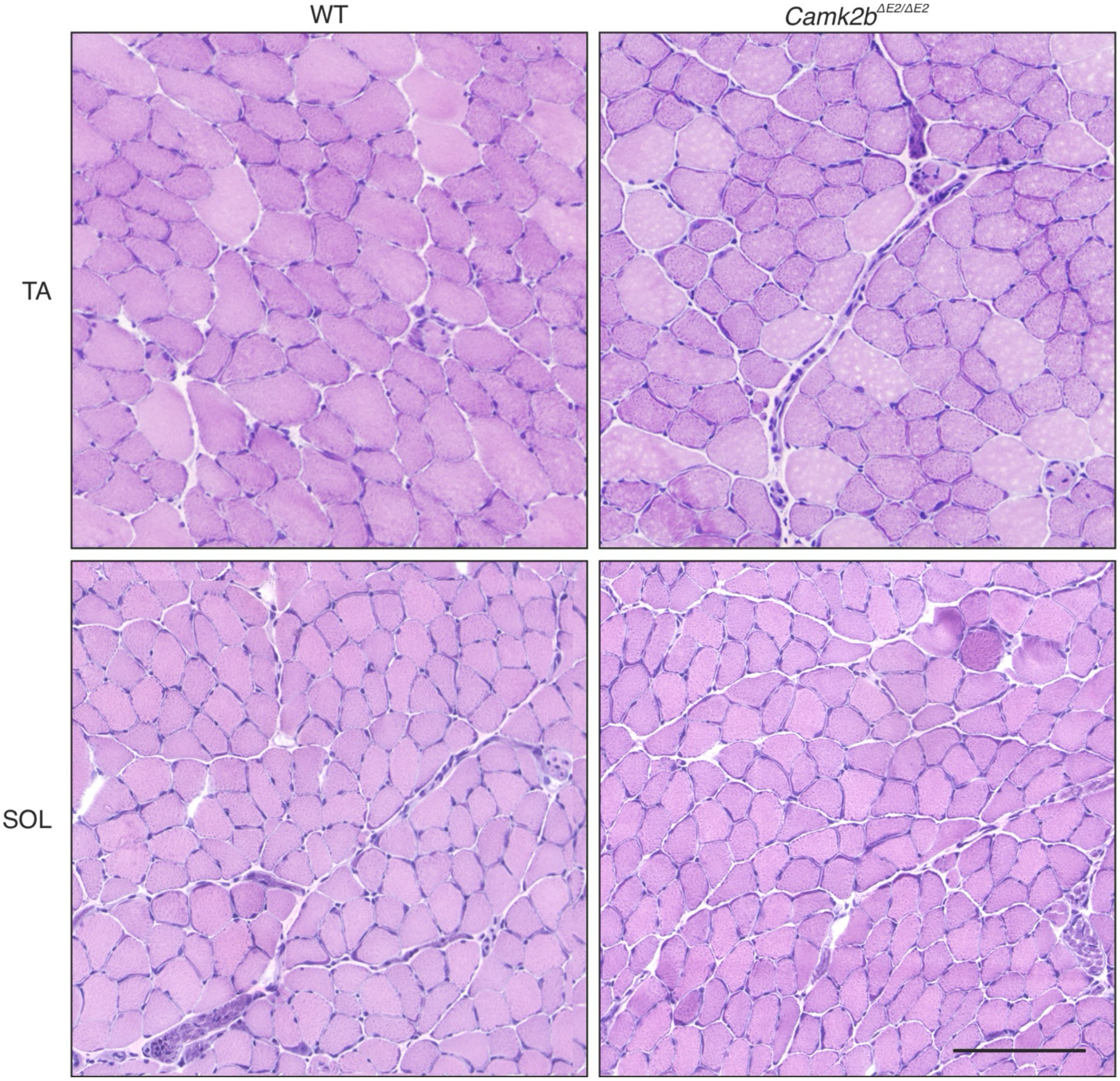

Supplementary Figure S3. Gross histology of *Camk2b*$^{-/-}$ animals did not exhibit obvious differences. *M. tibialis anterior* (TA) or *M. soleus* (SOL) of WT or *Camk2b*$^{-/-}$ animals were subjected to Hematoxylin-Eosin staining and assayed for center nucleated fibers as sign for muscle atrophy, immune cell infiltration or other obvious differences (n = 4). Images were acquired at a widefield microscope in brightfield using a 20x objective. Scale = 100 µm.

Raw Blots Fig. 2

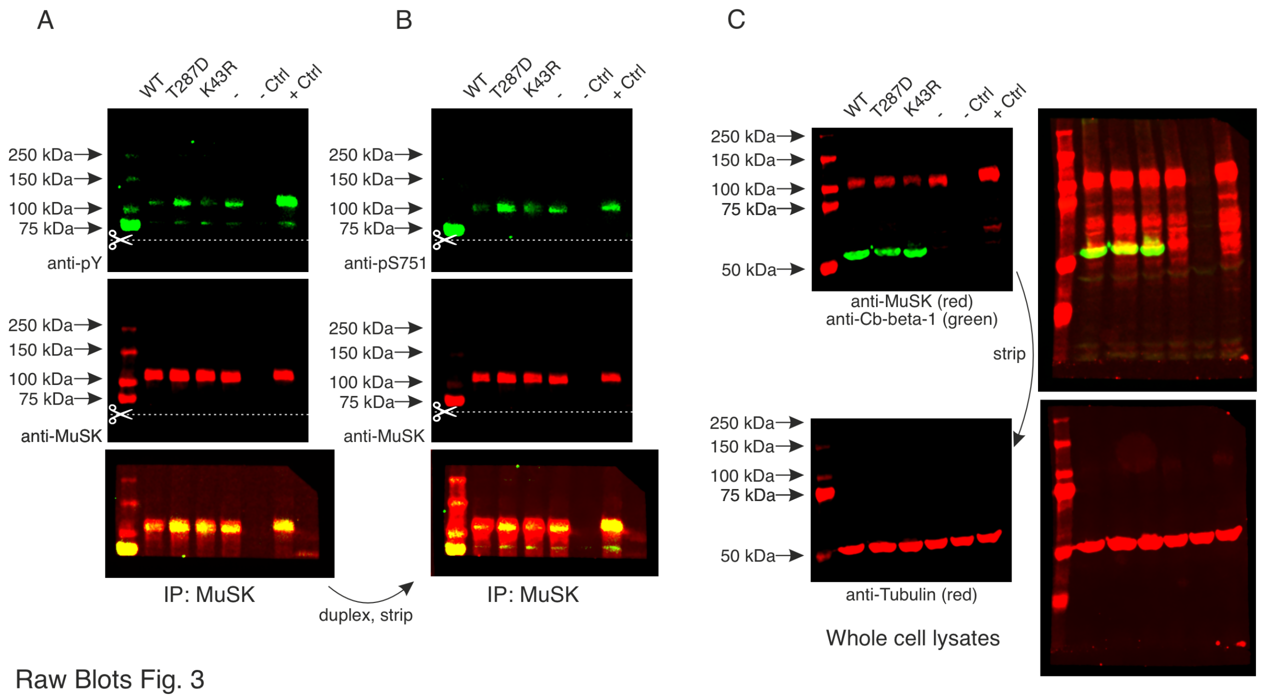

Raw Blots Fig. 3

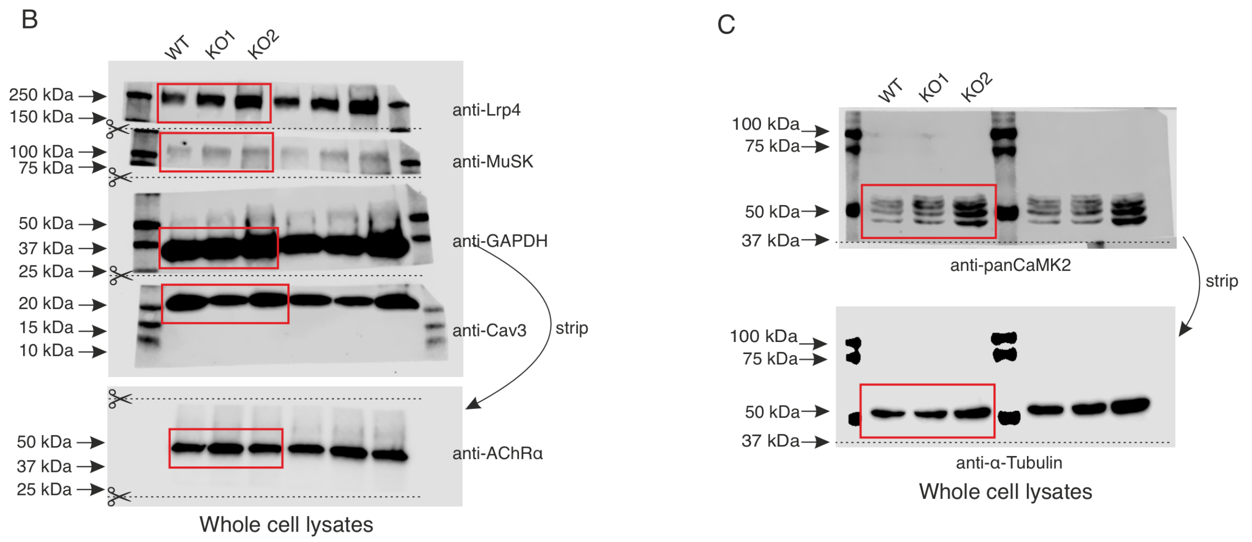

Supplementary Figure S4. Original Blots shown in Figures 2 and 3

Physical cuts are indicated with dashed lines and scissors. Position of molecular weight markers and direction of stripping are shown. Blot images in supplementary figures are adjusted to show membrane edges. (2A) In the case of MuSK-IPs, membrane was cut to remove IgGs. Merged blots show membrane edges. (2C) Merged blot shows membrane edges. (3B) Membrane was cut as indicated to enable simultaneous-probing with different antibodies, thereby avoiding stripping, which may reduce signal intensity and interfere with quantification.

Raw Blots Fig. 4

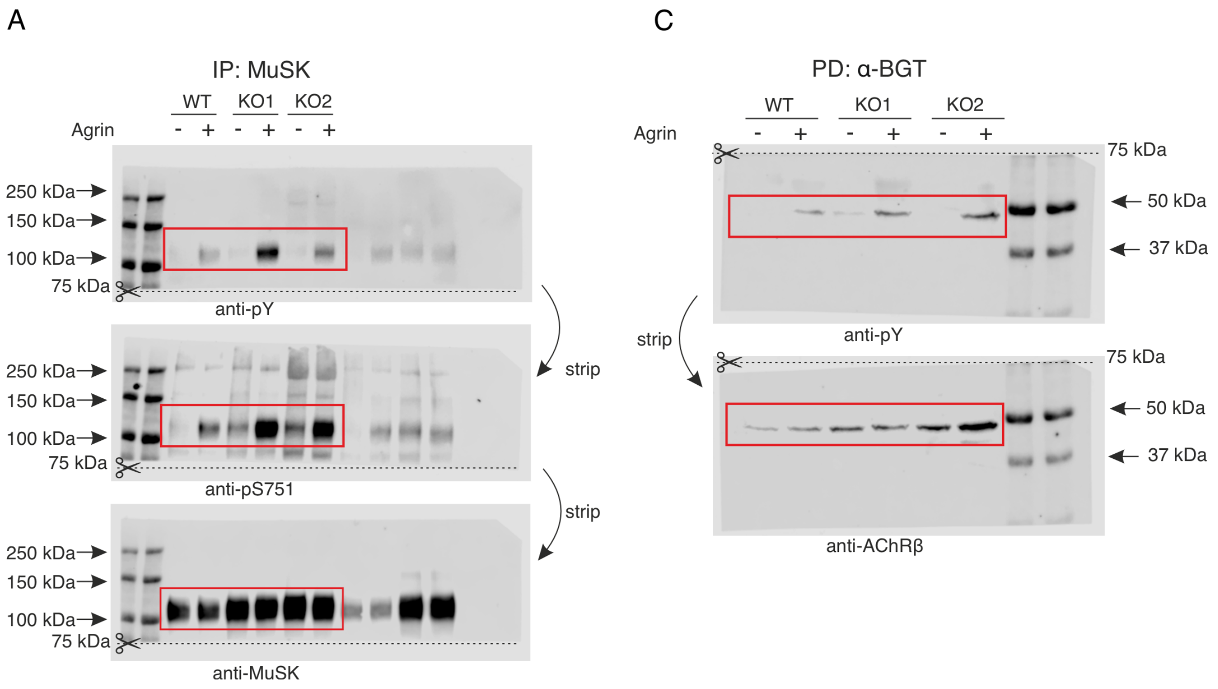

Supplementary Figure S5. Original Blots shown in Figure 4

Physical cuts are indicated with dashed lines and scissors. Position of molecular weight markers and direction of stripping are shown. Blot images in supplementary figures are adjusted to show membrane edges. (A) In the case of MuSK-IPs, membrane was cut to remove IgGs. (C) Membrane was cut just below 75 kD to remove the top part and allow antibody incubation in a smaller volume.

Raw Blots Fig. 5A

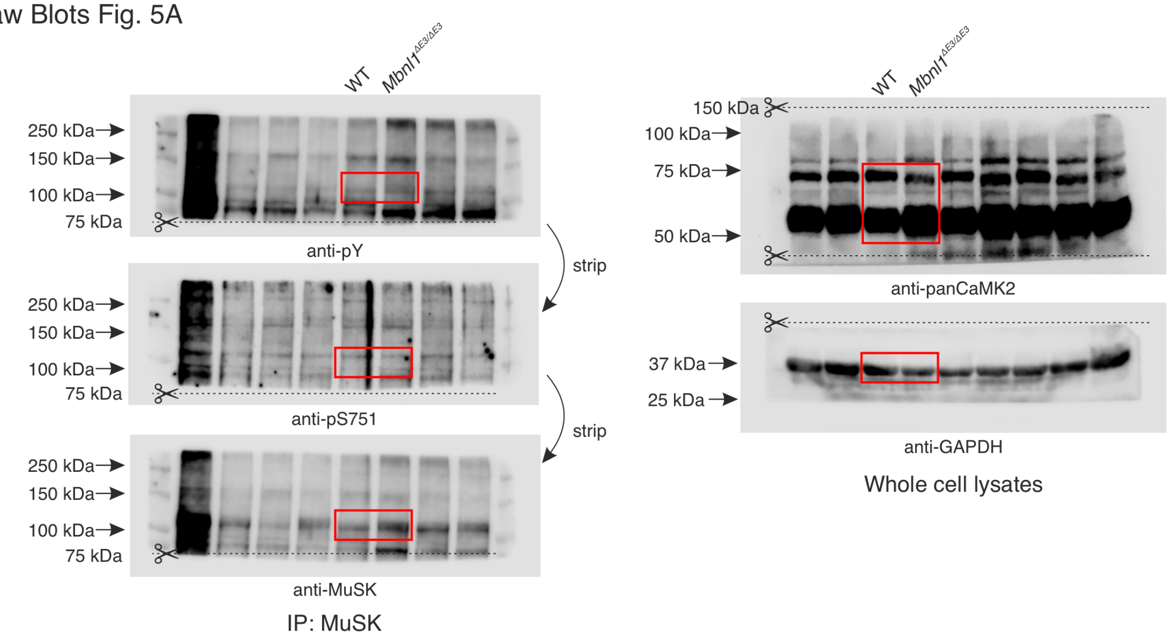

Raw Blots Fig. 6A

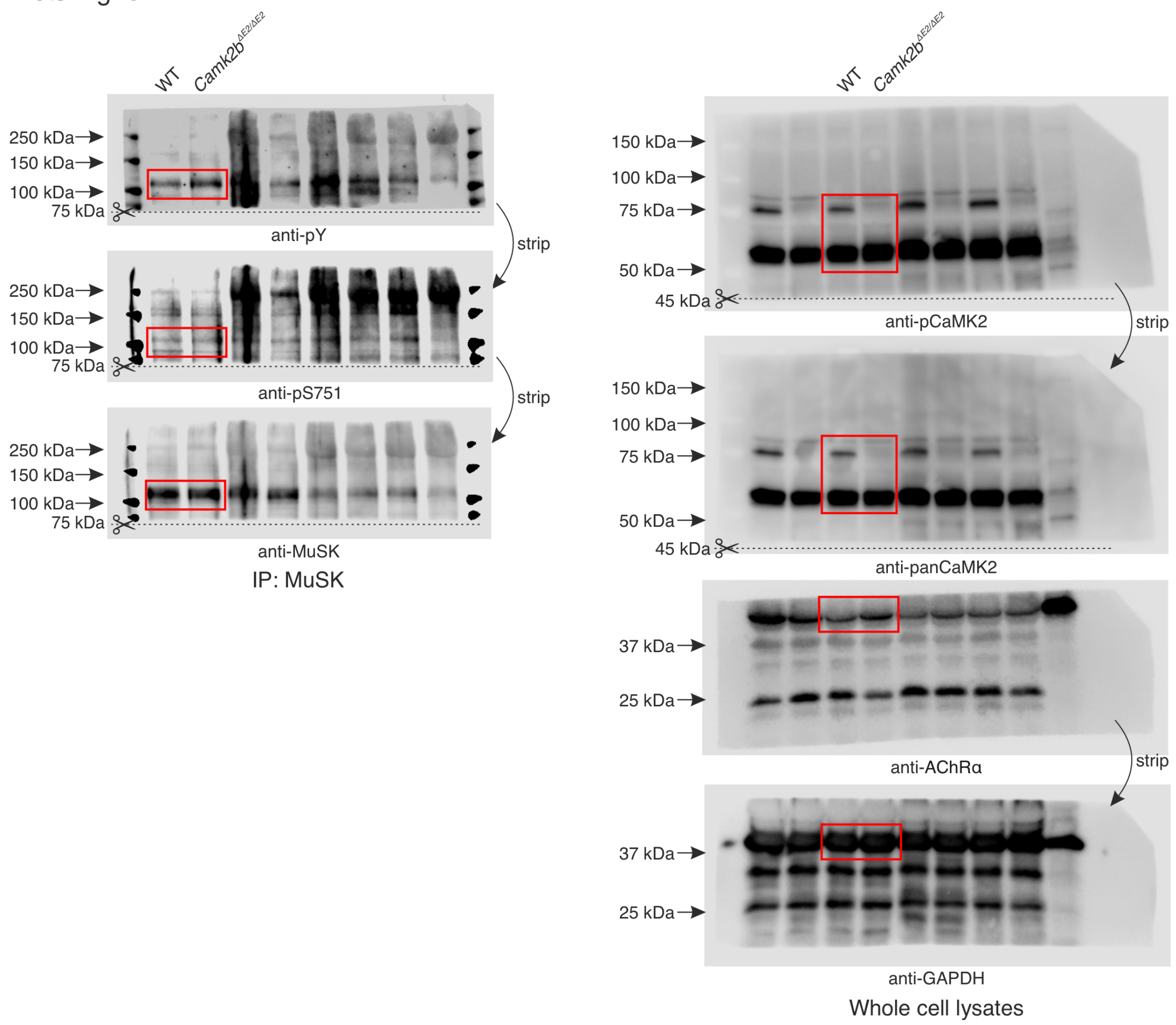

Supplementary Figure S6. Original Blots shown in Figures 5 and 6

Physical cuts are indicated with dashed lines and scissors. Position of molecular weight markers and direction of stripping are shown. Blot images in supplementary figures are adjusted to show membrane edges. In the case of MuSK-IPs, membrane was cut to remove IgGs. Membrane with whole cell lysates were cut as indicated to enable simultaneous-probing with different antibodies, thereby minimizing stripping, which may reduce signal intensity and interfere with quantification.
